# Combined inhibition of HMGCoA reductase and mitochondrial complex I induces tumor regression of BRAF inhibitor-resistant melanomas

**DOI:** 10.1186/s40170-022-00281-0

**Published:** 2022-02-22

**Authors:** Evelyn de Groot, Sruthy Varghese, Lin Tan, Barbara Knighton, Mary Sobieski, Nghi Nguyen, Yong Sung Park, Reid Powell, Philip L. Lorenzi, Bin Zheng, Clifford Stephan, Y. N. Vashisht Gopal

**Affiliations:** 1 Department of Melanoma Medical Oncology, University of Texas M.D. Anderson Cancer Center, Houston, TX USA; 2 Department of Translational Molecular Pathology, University of Texas M.D. Anderson Cancer Center, Houston, TX USA; 3 Department of Bioinformatics and Computational Biology, University of Texas M.D. Anderson Cancer Center, Houston, TX USA; 4 Institute of Bioscience and Technology, Texas A&M University, Houston, TX USA; 5 Cutaneous Biology Research Center, Massachusetts General Hospital and Harvard Medical School, Charlestown, MA USA

**Keywords:** Oxidative phosphorylation, Fatty acid metabolism, HMGCoA reductase, Statin, Melanoma, Therapeutic resistance

## Abstract

**Background:**

Primary and posttreatment resistance to BRAF^V600^ mutation–targeting inhibitors leads to disease relapse in a majority of melanoma patients. In many instances, this resistance is promoted by upregulation of mitochondrial oxidative phosphorylation (OxPhos) in melanoma cells. We recently showed that a novel electron transport chain (ETC) complex I inhibitor, IACS-010759 (IACS), abolished OxPhos and significantly inhibited tumor growth of high-OxPhos, BRAF inhibitor (BRAFi)–resistant human melanomas. However, the inhibition was not uniform across different high OxPhos melanomas, and combination with BRAFi did not improve efficacy.

**Methods:**

We performed a high-throughput unbiased combinatorial drug screen of clinically relevant small molecules to identify the most potent combination agent with IACS for inhibiting the growth of high-OxPhos, BRAFi-resistant melanomas. We performed bioenergetics and carbon-13 metabolite tracing to delineate the metabolic basis of sensitization of melanomas to the combination treatment. We performed xenograft tumor growth studies and Reverse-Phase Protein Array (RPPA)–based functional proteomics analysis of tumors from mice fed with regular or high-fat diet to evaluate *in vivo* molecular basis of sensitization to the combination treatment.

**Results:**

A combinatorial drug screen and subsequent validation studies identified Atorvastatin (STN), a hydroxymethylglutaryl-coenzyme A reductase inhibitor (HMGCRi), as the most potent treatment combination with IACS to inhibit *in vitro* cell growth and induce tumor regression or stasis of some BRAFi-resistant melanomas. Bioenergetics analysis revealed a dependence on fatty acid metabolism in melanomas that responded to the combination treatment. RPPA analysis and carbon-13 tracing analysis in these melanoma cells showed that IACS treatment decreased metabolic fuel utilization for fatty acid metabolism, but increased substrate availability for activation of the mevalonate pathway by HMGCR, creating a dependence on this pathway. Functional proteomic analysis showed that IACS treatment inhibited MAPK but activated AKT pathway. Combination treatment with STN counteracted AKT activation.

**Conclusions:**

STN and other clinically approved HMGCRi could be promising combinatorial agents for improving the efficacy of ETC inhibitors like IACS in BRAFi-resistant melanomas.

**Supplementary Information:**

The online version contains supplementary material available at 10.1186/s40170-022-00281-0.

## Background

Many cancers including melanomas acquire unique metabolic dependencies over their lifetimes [1–3]. These dependencies enable cancer cell survival in nutritionally challenging or newly metastasized environments or to counteract anticancer therapeutics. For example, activating mutations in BRAF or NRAS proteins hyperactivate the RAS/RAF/MEK/ERK MAP kinase (MAPK) pathway in melanoma cells, which increases glucose consumption and aerobic glycolysis [4]; and treatment with inhibitors targeting mutant-BRAF (BRAFi) or MEK (MEKi) inhibits glycolysis, forcing many melanomas to alter their metabolism towards increased mitochondrial oxidative phosphorylation (OxPhos) and utilize alternate fuels like fatty acids or glutamine [5–7]. Metabolism of these fuels is an important compensatory mechanism for the loss of glucose-dependent glycolytic activity and new reliance on mitochondrial OxPhos. Increased mitochondrial OxPhos promotes resistance to BRAFi/MEKi by also increasing anti-apoptotic mitochondrial signaling [8, 9]. Hence, targeting mitochondrial OxPhos is a promising therapeutic strategy against MAPK pathway inhibition-resistant cancers [10, 11].

The most well-known and well-tolerated OxPhos inhibitor is the antidiabetic drug, metformin, a weak ETC inhibitor that lacks adequate potency for inhibiting OxPhos and tumor growth. Its more potent analog Phenformin induces systemic toxicity in the context of diabetes treatment, but is currently being tested against various cancers in the clinic [12]. Among the newer agents, IACS-010759 (IACS), a potent ETC complex I inhibitor [13], completely abolishes OxPhos at low nanomolar doses and induces significant growth inhibition of some high OxPhos BRAFi-resistant, BRAF^V600^-mutant melanoma tumors, but weakly inhibits the growth of others with a similar metabolic phenotype, signifying the importance of specific dependencies for efficacy [14, 15]. Combination of IACS with BRAFi did not further improve antitumor activity of IACS in BRAFi/MEKi-resistant melanomas, whereas combination with MEKi induced systemic toxicity in mice [14]. Early phase I clinical trials with IACS in solid and hematological cancers showed partial responses, but with dose-limiting toxicity [16] (manuscript under preparation) (clinicaltrials.gov IDs: NCT03291938, NCT02882321). It is important to identify combination strategies that improve antitumor activity of IACS without increasing toxicity. In the current study, we performed an unbiased combinatorial drug screen in two BRAFi-resistant melanomas to identify such combinations with IACS. The screen identified molecularly dissimilar inhibitors exhibiting combination efficacy with IACS. In validation experiments, hydroxymethylglutaryl-coenzyme A (HMGCR) inhibitors exhibited higher potency compared with others and were chosen for comprehensive evaluation in this study.

## Methods

### Cell lines and inhibitors

The BRAFi- and MEKi-sensitive A375 human melanoma cell line was from ATCC, from which the BRAFi/MEKi-acquired resistant A375R1 was generated [9]. The intrinsic BRAFi and MEKi-resistant human melanoma cell lines UCSD354L, MEL624, and WM1799 were acquired from MDACC Cell Line Core. BRAF and MEK inhibitor sensitivities of the cell lines were previously reported [9, 17, 18]. Cell lines were authenticated by STR fingerprinting, and all cells were grown in RPMI media containing 5% fetal bovine serum. IACS-010759 (IACS) was developed and synthesized at the MDACC Institute for Applied Cancer Science as described [13]. Compounds in the drug screen obtained from the Selleck L2000 customized bioactive compound library (Selleck Chem) consisted of 320 therapeutic agents or preclinical candidates with a wide variety of target specificities (Table S1). For *in vitro* treatments, all compounds were dissolved in DMSO. For *in vivo* treatments, suspensions of the compounds were prepared using the following vehicles: 0.5% methyl cellulose for IACS-010759 (IACS) and 35% PEG300 + 2% Tween-80 for Atorvastatin (STN).

### Combinatorial drug screen

**Fig. 1:**
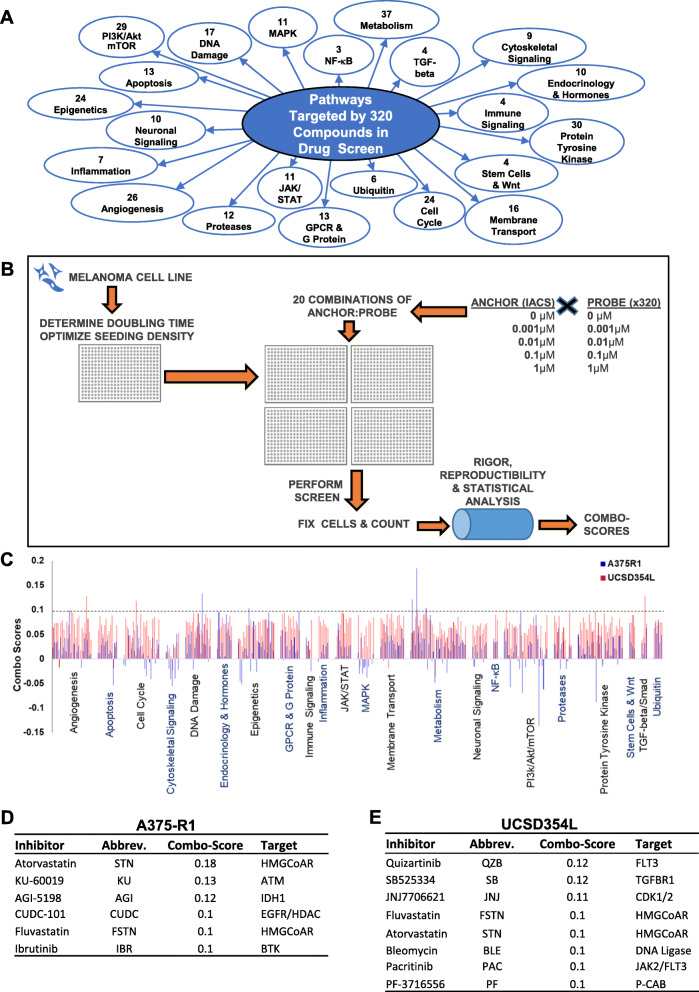
High-throughput combinatorial drug screen. **A** The 320 compounds (probes) in the drug screen consist of clinically active or preclinical agents that target 22 broad biological pathways shown. **B** Schematic of the drug screen showing growth optimization of A375R1 and UCSD354L cells, followed by testing of five combinations of tenfold concentrations of anchor (IACS) and each of the 320 probes on these cells, counting cells after 72 h, and lastly an analytical pipeline to generate combo scores. **C** Combo scores shown as bar graphs, with a horizontal dashed line representing cutoff scores for synergy (> 0.1). **D** & **E** Probes that exhibited the highest synergistic inhibition of A375R1 and UCSD354L cells respectively in combination with IACS

The drug screen was performed at the High Throughput Research and Screening Center, Institute of Bioscience and Technology, Texas A&M University, following the methodology depicted schematically in Fig. 1A. Briefly, optimal seeding densities that maintain log-phase growth of melanoma cell lines over a 96-h assay window were determined using a cell growth curve analysis with Hoescht-33342 nuclear staining. This was followed by seeding of optimal cell numbers of each cell line and treatments with either the anchor compound (IACS), individual probe compounds, or their combinations with the anchor compound using 10-fold dose dilution ranges. Cell growth inhibition induced by the treatments over a period of 72 h was assessed using DAPI nuclear staining. The results were subjected to rigor and reproducibility analysis, and synergistic or additive effects of the combinations were determined using a bootstrapped bliss independence model. Detailed methodology of the drug screen is provided in the supplemental methods.

### Cell biological studies

Cell proliferation inhibition was evaluated using Cell Titer Blue (Promega) or 0.5% crystal violet staining after 72 h inhibitor treatments of cells grown in 96-well plates. IC50 values of serially diluted inhibitor-induced cell growth inhibition were determined by nonlinear regression curve analysis in the GraphPad Prizm software. Cell death was determined by Propidium iodide–cell cycle analysis using flow cytometry and cytoplasmic histone-associated DNA fragment analysis using Cell Death Detection ELISA Plus kit (Roche Applied Science) as described previously [17].

### Seahorse metabolic flux analyses

Mitochondrial stress tests were performed to determine real-time oxygen consumption rate (OCR) and extracellular acidification rate (ECAR) in melanoma cell lines as described earlier [9]. Final assay concentrations of inhibitors used in the mitochondrial stress test were 1.5 μM oligomycin, 0.5 μM FCCP, 0.5 μM rotenone, and 0.5 μM antimycin A. Metabolic Fuel Flex tests were performed to evaluate glucose, glutamine, and fatty acid dependencies of melanoma cell lines. Final assay concentrations of inhibitors used in this test were 3 μM BPTES, 4 μM etomoxir, and 2 μM of UK5099. The metabolic flux tests were performed in 96-well plates (2 × 10^4^ cells/well) using a Seahorse XFe96 analyzer, and data was normalized against cell numbers following the manufacturer’s protocol (Agilent Technologies).

### Stable ^13^C isotope tracing analysis of glucose and glutamine

Melanoma cells (3 × 10^6^ cells/150-mm dish) were seeded overnight, labeled with [U-^13^C]-glucose or [U-^13^C]-glutamine and treated with the indicated inhibitors for 12 h. [U-^13^C]-glucose and [U-^13^C]-glutamine tracing analyses were performed at the MDACC Metabolomics Core Facility as described before [7]. Detailed methodology is described in the supplemental methods.

### Protein analysis

Whole cell lysates from cell lines were prepared in RIPA lysis buffer, and protein lysates from tumors were prepared by homogenization of ~ 50 mg of tumor tissue in a bead homogenizer as previously described [14]. The protein lysates were denatured and used for western blotting using standard methods, or for Reverse-Phase Protein Array (RPPA) analysis at the MDACC Functional Proteomics Core Facility, and the data was analyzed as described previously [9]. Antibodies used for western blotting and RPPA are listed at the RPPA core website (www.mdanderson.org/research/research-resources/core-facilities/functional-proteomics-rppa-core.html). RhoA (67B9) antibody was from Cell Signaling Technology.

### *In vivo* xenograft growth studies

Subcutaneous xenograft tumors were generated for A375R1 and UCSD354 cell lines with 3 × 10^6^ cells/animal in the right flank of NOD scid gamma (NSG) mice. The mice were separated into two diet cohorts—regular diet (caloric profile of 62.1% carbohydrate, 24.7% protein, 13.2% fat) (PicoLab #5053), and high-fat ketogenic diet (caloric profile of 1.8% carbohydrate, 4.7% protein, 93% fat) (Bio-Serv #F3666). Inhibitor treatments were performed by oral gavage daily once for the indicated number of days. Tumor volumes and mice weight were recorded every 3 days. Animals were excluded if they showed overt toxicity or lost > 15% body weight over the treatment course. For molecular analysis of inhibitor effects, tumor-bearing mice were treated with the inhibitors as above, and tumors were harvested 3 h after drug administration on the second day of treatment. All animal experiments were approved by the Institutional Animal Care and Use Committee.

### Statistical analysis

Rigor and reproducibility of the combinatorial drug screening assay was performed in accordance with the NCATS Assay Guidance Manual [19], and a bootstrapped bliss independence model was used to calculate drug synergy [20]. *In vivo* tumor growth studies consisted of nine mice per treatment group which provided significant power for tumor growth analysis. For functional proteomics analysis using RPPA, tumors were harvested from mice after 5 days of treatments, and protein lysates from three pieces of each tumor were analyzed separately. Hierarchical supervised clustering of significantly differing proteins in treated versus untreated samples was performed using Pearson correlation in Gene Cluster 3.0, and heatmaps were generated using Gene Treeview. For *in vitro* cell proliferation assays, significant differences between treatments and individual doses of treatments were analyzed by two-way ANOVA followed by post hoc Tukey multiple comparisons test in GraphPad Prizm. For tumor growth studies, cell death assays, metabolite and RPPA analyses, T tests were used for determining statistically significant differences (*p* < 0.05 for cell death and tumor growth studies, *p* < 0.005 for RPPA) between inhibitor treatments and mock/vehicle treatments. Where necessary, significant differences from mock/vehicle treatments or between treatments were designated with asterisks (*).

## Results

### Combinatorial drug screen identified compounds that improved melanoma cell growth inhibition by IACS-010759 (IACS)

For identifying clinically relevant small molecules that significantly improve anti-melanoma activity of IACS, we performed an unbiased combinatorial drug screen that tested the combination of IACS (anchor) with each of 320 inhibitors (probes) that target 22 different signaling pathways (Fig. 1A and Table S1). We tested these combinations in two *BRAF*^*V600E*^-mutant melanoma cell lines, UCSD354L and A375-R1, which possess intrinsic and acquired resistance respectively to BRAF/MEK inhibition [9, 17, 18]. The drug screen was performed as shown schematically in Fig. 1B, and cell growth inhibition induced by each probe and their combinations with IACS was determined after 72 h of incubation. Combination efficacy was determined using a bootstrapped bliss independence model and represented as “combo score” units, with ≥ 0.1 representing potentially synergistic growth inhibition. Values under 0.1 represent additive effects, and negative values potentially antagonistic effects. The combo scores revealed that combination with IACS induced mostly additive effects on cell growth inhibition by a majority of the 320 probes in both cell lines, with less than 20 probes showing antagonism (Fig. 1C). Less than ten probes showed potentially synergistic growth inhibition when combined with IACS in either cell lines (Fig. 1D and E).

### Combination of IACS with hydroxymethylglutaryl coenzyme A (HMGCoA) reductase inhibitors induced potent growth inhibition of BRAFi-resistant melanomas

**Fig. 2:**
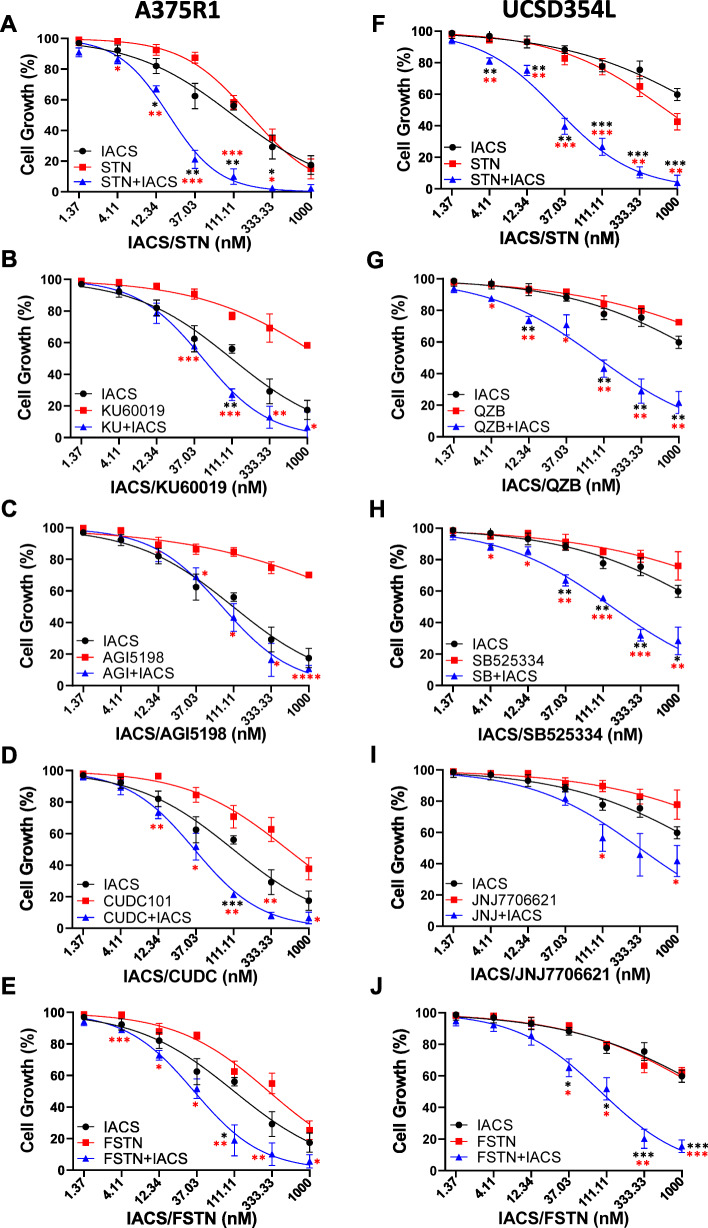
Cell growth inhibition by treatment combinations that showed synergistic combo scores. **A**–**E** A375R1 cells were seeded in 96-well plates (10^3^ cells/well) and treated with individual probes and their combinations with IACS at 1:1 concentration shown on the x-axes. Cell growth inhibition was determined after 72 h using Cell Titer Blue reagent. **F–J** Same experiment as above, conducted on UCSD354L cells. Data is normalized to vehicle-treated cells and is average of triplicates, with error bars representing standard deviation (*SD*), and colored asterisks representing significant differences (*< 0.033, **< 0.002, and ***< 0.001) in effects for combination treatment versus individual probes (red) or IACS (black)

The combinatorial drug screen results were validated with five probes that exhibited the highest combo scores with IACS in each of the two cell lines. Two probes with non-synergistic combo scores of < 0.1 were also tested to validate their lower efficacy observed in the screen. The cell lines were seeded in 96-well plates and treated with the indicated dose ranges of single agents or their combinations with IACS, and cell growth inhibition after 72 h was assessed using the Cell Titer Blue cell proliferation assay; IC50 values were determined. The results showed that among the various probes tested, the HMGCoA reductase inhibitor (HMGCRi), atorvastatin (STN), exhibited the most potent combination effect with IACS in both cell lines (Fig. 2A for A375R1 and Fig. 2F for UCSD354L). Compared with STN, other high-scoring probes from the screen exhibited lower combined efficacy with IACS (Fig. 2B–E for A375R1 and Fig. 2G–J for UCSD354L). Two non-synergistic probes from the screen, GSK690693 and BKM120, exhibited lower combined efficacy with IACS compared to IACS + STN in a separate validation study (Fig. S1A-B). IC50 values for all individual treatments and their combinations with IACS are shown in Table S2. We also evaluated the highest scoring IACS + STN in normal melanocytes and found that neither single agent nor their combination significantly inhibited the growth of these normal cells (Fig. S1C). Finally, to confirm that the Cell Titer Blue cell proliferation assay does not produce false-positive growth inhibitory effects, we also performed crystal violet staining of IACS + STN–treated cells and found a similar growth inhibitory profile as seen with Cell Titer Blue (Fig. S1D). Taken together, the drug screen and subsequent validation studies suggest that the HMGCRi, STN, could be a potent combination treatment for improving the efficacy of IACS treatment against BRAFi/MEKi-resistant, BRAF-mutant melanomas.

### Cellular and molecular effects of STN + IACS treatments on melanoma cells

**Fig. 3:**
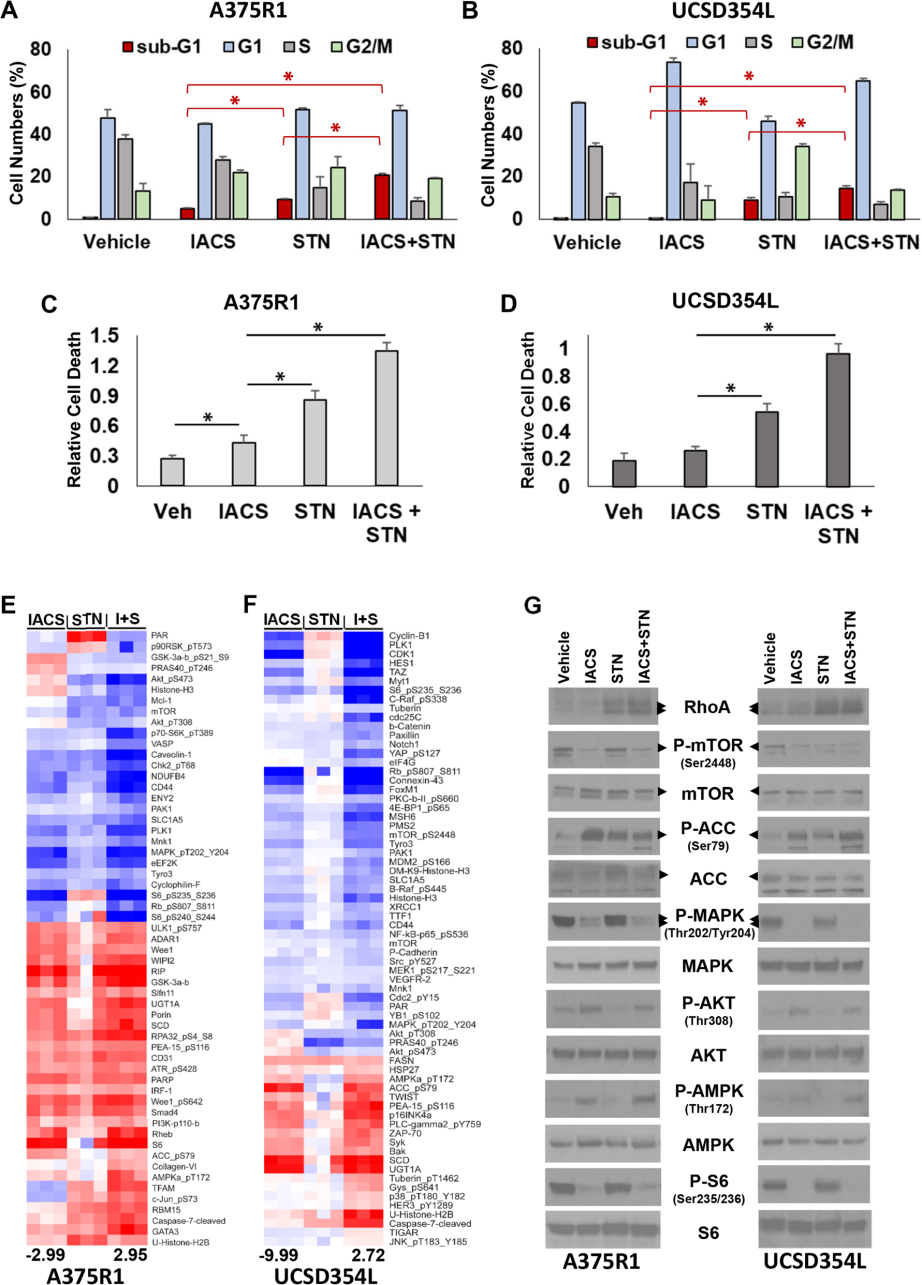
In vitro and in vivo efficacy of STN + IACS combinations. **A** and **B** A375R1 and UCSD354L cells were treated with IACS (100 nM) or STN (1 μM) or their combinations for 72 h, and cell cycle profiles were generated using propidium iodide–FACS analysis, which included sub-G1 (dead cell) population. **C** and **D** Relative cell death induced by the same treatments was confirmed using Cell Death ELISA assay. Data in A–D are in triplicates; error bars, *SD*. **E** and **F** Supervised clustering heatmaps of RPPA-analyzed proteins from A375R1 (**E**) and UCSD354L (**F**) that were significantly (*p* < 0.005) altered after 24 h treatment of cells with IACS, STN, or their combination. The heatmap represents ratio of results from inhibitor-treated versus vehicle-treated samples. Intensity ranges of lowest (blue) and highest (red) protein levels are indicated at the bottom of the heatmaps. **G** Western blotting of representative proteins and phosphoproteins detected by the RPPA analysis

As STN exhibited the most potent anti-melanoma combination efficacy with IACS among all probes with synergistic combo scores, we further explored the efficacy of this combination on melanoma cell death induction. Propidium iodide cell cycle analysis showed that a 72 h IACS treatment of A375R1 induced a small increase of G2/M and sub-G1 phase cells. STN treatment induced small increases of G2/M phase and a significant increase of sub-G1 phase, indicating cell death induction by the treatment, which further increased following IACS + STN treatment (Fig. 3A). In the UCSD354L, IACS induced G1 arrest and STN induced G2/M arrest, while the combination induced G1 arrest and sub-G1 accumulation (Fig. 3B). Combination of the standard of care BRAFi, dabrafenib (DAB), with IACS did not induce sub-G1 accumulation in either cell line, although DAB + STN induced a small, but insignificant increase compared with STN alone in A375R1, but not in UCSD354L (Figs. S1A and S1B). Cell death induction by IACS and STN combination treatments in both cell lines was confirmed using a cytoplasmic histone accumulation assay that sensitively detects cell death as increased cytoplasmic accumulation of histones in treated versus untreated cells (Fig. 3C and D).

For identifying the molecular determinants of sensitivity in the BRAFi-resistant melanomas in our study, we extracted protein lysates from the cells after 24 h treatments with 100 nM IACS, 1 μM STN, or their combinations and performed Reverse-Phase Protein Array (RPPA) analysis to assess functional status of over 200 cellular proteins in key oncogenic signal transduction pathways. We performed Pearson correlation and supervised clustering of the most significant (*p* < 0.005) treatment-induced alterations compared with vehicle treatments, and generated heatmaps of the treatment ratios for both cell lines (Fig. 3E and F). These results showed that IACS treatment activated phospho-AMPK_Thr172 and phospho-ACC_Ser79, inhibited phospho-S6_ Ser235_236_240_244 and phospho-MAPK_Thr202_Tyr204, as has been shown previously by us and others as targeted effects of inhibiting mitochondrial activity [14, 21, 22]. Interestingly in both cell lines, IACS treatment induced phospho-AKT_Thr308/Ser473, which is known to promote melanoma resistance to BRAFi/MEKi [23, 24]. Treatment with STN decreased phospho-AKT compared with vehicle treatment and also counteracted its IACS-induced increase in both cell lines. IACS + STN combination also downregulated pro-growth signaling proteins like phospho-Rb_Ser807_811 and upregulated growth inhibitory/cell death proteins (for example, cleaved caspase 7) (Fig. 3E and F). Some of the key IACS + STN–induced alterations revealed by RPPA were confirmed by western blotting analysis of phospho- and total proteins in protein lysates (Fig. 3G). RhoA protein upregulation was assessed as a marker of HMGCR inhibition by STN. Treatment-induced alterations in the levels of phospho-AKT_Thr308 and phospho-AMPK_Thr172 were quantified using NIH Image J software and represented as bar graphs (Fig. S1G-J).

### Metabolic effects of STN + IACS combination in melanoma cells

**Fig. 4:**
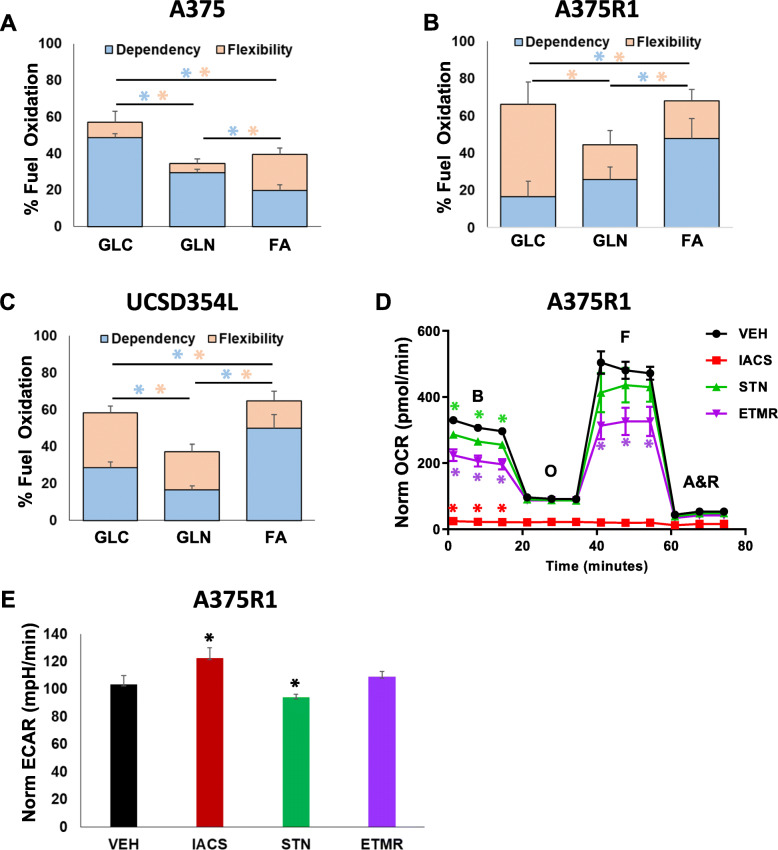
Metabolic features and effects of IACS and STN treatments. **A**, **B**, **C** The Seahorse Fuel Flex assay was performed on the A375, A375R1, and UCSD354L cells to determine their dependency (blue) on glucose (GLC), glutamine (GLN), and fatty acids (FA), and their flexibility (orange) to utilize either of the single nutrients when the other two are inhibited. Data are in quadruplicates; error bars, SD. **D** Seahorse extracellular flux analysis in A375R1 cells. Basal (“B”), oligomycin-inhibited (“O”), FCCP-activated (“F”), and antimycin and rotenone-inhibited (“A&R”) OCR levels were determined after treatments with either vehicle, 100 nM IACS, 1 μM STN, or 1 μM ETMR for 24 h. **E** Treatment-induced alterations in basal ECAR levels in the cells from the experiment in *D*. Data represents cell numbers—normalized quadruplicates; and error bars, *SD*

The above protein analysis revealed that IACS + STN treatment induced significant alterations in phosphoproteins associated with mitochondrial metabolism like AMPK and ACC. STN inhibits the conversion of HMG-CoA to mevalonate, the first and rate-limiting step of cholesterol biosynthesis pathway [25, 26]. As the levels of HMG-CoA are dependent on acetyl CoA, a critical node in mitochondrial metabolism, we hypothesized that responsiveness to IACS + STN may be associated with mitochondrial metabolism. To test that, we performed the Seahorse Fuel Flex assay in parental BRAFi-sensitive A375 cells, BRAFi-acquired resistant A375R1 and intrinsic BRAFi-resistant UCSD354L cells. In this assay, we assessed the dependency of the cells on each of the three cellular fuels—glucose (GLC), glutamine (GLN), or fatty acids (FA)—and their flexibility to use either of the single fuels when the other two fuels are inhibited. The results showed that the parental BRAFi-sensitive A375 cells have the highest dependency on GLC (Fig. 4A), whereas the two BRAFi-resistant cells have the highest dependency on FA (Fig. 4B and C). A375 possessed low flexibility for compensatory utilization of any two alternate fuels when one fuel was inhibited, although the cells did possess as much capacity to oxidize FA as their basal dependency on this fuel (Fig. 4A). In comparison, the two resistant cell lines showed a comparatively higher flexibility to oxidize any single fuel when the other two were inhibited, with the highest flexibility for GLC utilization (Fig. 4B and C). The higher FA oxidation dependency of the two BRAFi-resistant melanoma cells indicates their altered metabolic requirement for FA compared with the sensitive cells and may influence their responses to metabolism targeting therapeutics. Two other BRAFi/MEKi-resistant cells that did not exhibit FA dependency were not sensitive to IACS + STN combination treatment (Fig. S2A-S2D).

Previous studies showed that statins interfere with calcium homeostasis to inhibit mitochondrial complexes I and III [27], and can weakly inhibit OCR in cancer cells [28]. To determine if STN could inhibit OCR in the BRAFi-resistant melanoma cells in our study, we performed Seahorse bioenergetics stress tests in A375R1 cells. The results showed that 24 h treatment with 1 μM STN minimally inhibited basal cellular oxygen consumption rate (OCR), interpreted as an indirect readout for cellular OxPhos (Fig. 4D). STN also induced a small but significant inhibition of basal extracellular acidification rate (ECAR), an indirect readout for glycolytic activity (Fig. 4E). On the other hand, treatment with 100 nM IACS completely inhibited basal and maximal OCR and slightly increased ECAR levels as shown previously [14]. As A375R1 cells show elevated FA dependence, we also tested etomoxir (ETMR), a carnitine palmitoyltransferase inhibitor that inhibits FA β-oxidation. ETMR induced significant inhibition of basal and maximal OCR compared with STN. However, unlike STN, it did not inhibit A375R1 cell proliferation (Fig. S2E). Taken together, these results suggest that the weak OCR inhibition by STN is unrelated to its cell growth inhibition. As IACS treatment induced a complete inhibition of OCR, with no increase from FCCP treatment, we replicated the above IACS and the mitochondrial inhibitor treatments in a separate 96-well plate and assessed cell viability at the end of each subsequent treatment using trypan blue dye exclusion. The results showed < 5% decrease of viability in IACS-treated cells following FCCP treatment, suggesting that the complete OCR inhibition by IACS was not a result of altered cell viability (Fig. S2F).

**Fig. 5:**
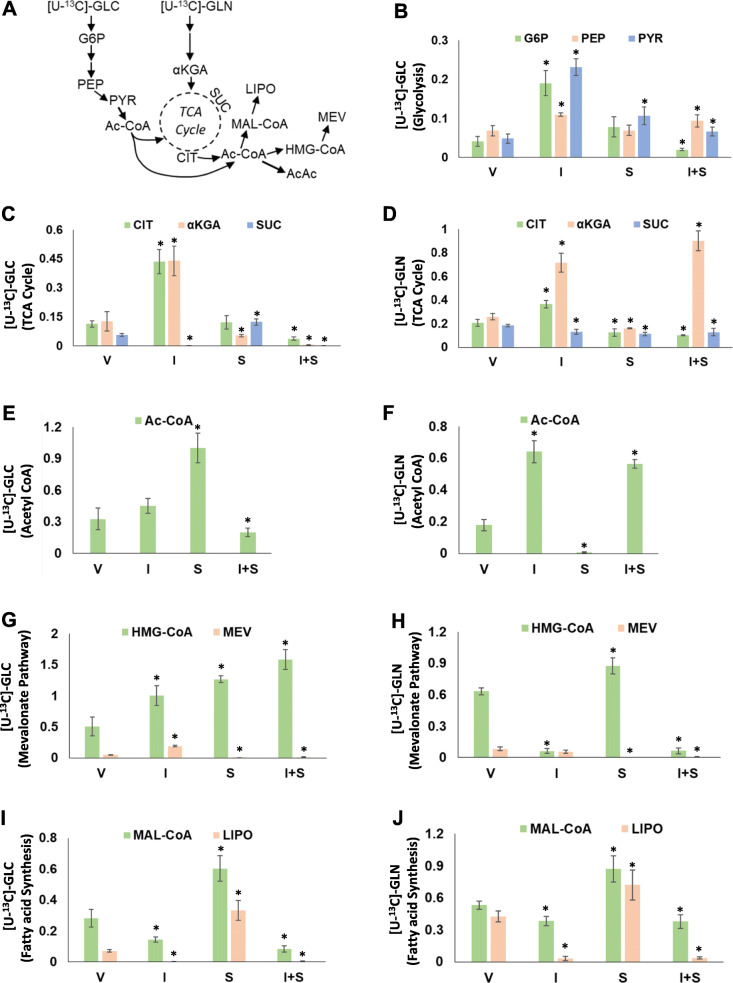
[^13^C]-labeled metabolite tracing analysis. **A** Schematic showing metabolites labeled by [U-^13^C]-glucose (GLC) or [U-^13^C]-glutamine (GLN) after treatment of A375R1 cells with vehicle (V), IACS (I), STN (S), or their combination (I + S) for 12 h. **B** Fractional labeling of glycolysis pathway metabolites glucose-6-phosphate (G6P), phosphoenolpyruvate (PEP), and pyruvate (PYR) by [U-^13^C]-GLC (glucose) following treatment with IACS (I) and STN (S) or their combination (I + S). **C** and **D** Fractional labeling of TCA cycle metabolites, citrate (CIT), α-ketoglutarate (αKGA), and succinate (SUC) by [U^13^C]-GLC (**C**) and [U-^13^C]-GLN (glutamine) (**D**). **E** and **F** Fractional labeling of acetyl CoA (Ac-CoA) by [U^13^C]-GLC (**E**) and [U-^13^C]-GLN (**F**). **G** and **H** Fractional labeling of HMG-CoA and mevalonate (MEV) by [U^13^C]-GLC (**G**) and [U-^13^C]-GLN (**H**). **I** and **J** Fractional labeling of malonyl CoA (Mal-CoA) and lipoic acid (LIPO) by [U^13^C]-GLC (**I**) and [U-^13^C]-GLN (**J**). Y-axis represents normalized relative fractional abundances of ^13^C-isotopologues, and x-axis represents indicated treatments. Data is average of triplicates and error bars *SD*

We then performed ^13^C-labeled GLC and GLN tracing analyses to mechanistically assess the above molecular and metabolic effects of IACS and STN, and to potentially identify a metabolic basis for melanoma cell growth inhibition by their combination. We treated A375R1 cells grown in [U-^13^C]-GLC or [U-^13^C]-GLN media with 100 nM IACS, 1 μM STN, or their combination for 12 h and detected relative incorporation of ^13^C-labeled metabolites in glycolysis, tricarboxylic acid cycle (TCA cycle), FA synthesis, and mevalonate/HMGCoA pathways using LC-MS (Fig. 5A). The results showed that IACS (I) treatment stimulated relative GLC incorporation into the glycolysis metabolites—glucose 6 phosphate, phosphoenolpyruvate, and pyruvate (G6P, PEP, and PYR)—compared to vehicle (V), while STN (S) induced a small increase and IACS + STN (I + S) inhibited incorporation into G6P, suggesting that the combination treatment inhibits the initial step of glycolysis (Fig. 5B). Similar effects were observed in the TCA cycle, where IACS stimulated relative GLC and GLN incorporation into citrate and α-ketoglutarate (CIT and αKGA), and combination with STN inhibited these effects (Fig. 5C and D). Interestingly, STN increased relative GLC incorporation into acetyl CoA (Ac-CoA), while IACS increased relative GLN incorporation into Ac-CoA (Fig. 5E and F), which reveal the unique metabolic effects of the two inhibitors. Also, interestingly, IACS treatment increased relative GLC incorporation but inhibited relative GLN incorporation into HMGCoA, the substrate for HMGCR enzyme in the mevalonate pathway (Fig. 5G and H). STN treatment induced relative accumulation of GLC and GLN carbons in HMGCoA, an expected effect of substrate accumulation in the wake of HMGCR inhibition, and a corresponding decrease in mevalonate (MEV) (Fig. 5G and H). Combination treatments inhibited incorporation of GLN into HMG-CoA and also inhibited GLC and GLN incorporation into MEV (Fig. 5G and H). Finally, treatment-induced alterations in the relative incorporation of GLC and GLN into malonyl CoA (MAL-CoA) and its downstream fatty acid product, lipoic acid (LIPO), suggest that IACS treatment inhibits FA biosynthesis, STN increases it, and the combination inhibits it (Fig. 5I and J). As an earlier study showed that upregulation of acetoacetate (AcAc), a ketogenic metabolite of Ac-CoA promotes the growth of BRAF^*V600E*^ tumors [29], we evaluated treatment effects on relative GLC and GLN carbon incorporation into AcAc. The results showed that IACS and IACS + STN potently inhibited relative GLC incorporation into AcAc, while IACS induced a small decrease of relative GLN incorporation (Fig. S2H and I). The individual and combination treatments induced an increase of total pooled Ac-CoA levels (Fig. S2G). Taken together, these results suggest that although individually, IACS and STN have differential effects on cellular utilization of GLC and GLN; their combination ultimately inhibits relative incorporation of the two fuels into FA synthesis and mevalonate pathways.

### IACS + STN combination induces regression of melanoma tumor growth

**Fig. 6:**
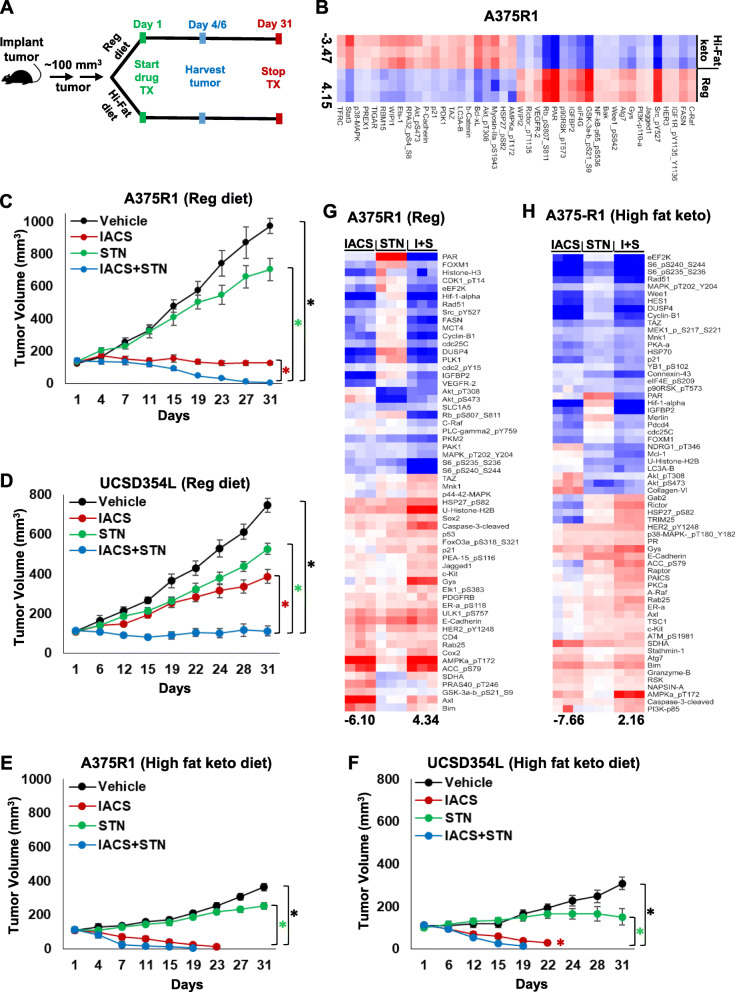
Tumor xenograft growth studies. **A** Schematic showing subcutaneous tumor growth analysis in mice fed with regular (Reg) or ketogenic high-fat (Hi-Fat) diet. Mice were treated daily once with vehicle, 5 mg/kg IACS, 1 mg/kg STN or their combinations for the number of days shown in (**C**–**F**). Tumor harvests for molecular studies were performed on the fourth (A375R1) or sixth (UCSD354L) day of treatments. **B** Heatmap showing unsupervised clustering analysis of significantly (*p* < 0.005) altered proteins in A375R1 tumors harvested from mice fed with regular (Reg) or high-fat keto diet and analyzed by RPPA. Heatmap represents Pearson correlation of significantly (*p* < 0.005) different protein levels in tumors from mice fed with high-fat keto (Hi-fat keto) versus regular (Reg) diet. Intensity ranges of lowest (blue) and highest (red) protein levels are indicated at the bottom of the heatmap. **C** and **D** A375R1 (**C**) and UCSD354L (**D**) tumor growth inhibition by IACS and STN treatments in mice fed with regular (Reg) diet. **E** and **F** A375R1 (**E**) and UCSD354L (**F**) tumor growth inhibition by IACS and STN treatments in mice fed with high-fat keto diet. Tumor volumes were recorded on the days shown on the x-axis, and tumor growth was represented as line graphs. Tumor growth data is from eight mice/group, error bars, standard error of mean (SEM). **G** and **H** Supervised clustering analysis of treatment-induced alterations of RPPA-analyzed proteins in tumors from mice fed with Reg (**G**) and Hi-Fat keto (**H**) diets. Heatmaps show Pearson correlation of significantly (*p* < 0.005) altered protein levels presented as ratios of inhibitor-treatments versus vehicle-treatments. Intensity ranges of lowest (blue) and highest (red) protein levels are indicated at the bottom of the heatmaps

We assessed *in vivo* effects of the individual and combination treatments in subcutaneous A375R1 and UCSD354L tumors in mice fed with normal carbohydrate-rich chow (62% calories from carbohydrate) (Fig. 6A). Additionally, as our above results suggested that these tumor cell lines have FA fuel dependency and that IACS + STN treatments potently inhibit lipid metabolism, we functionally assessed the effect of the treatments on tumor growth in mice fed with a high-fat ketogenic diet (93% calories from fat) (Fig. 6A). RPPA proteomics analysis of protein lysates from untreated mice with A375R1 (Fig. 6B) and UCSD354L (Fig. S3A) tumors showed that in comparison with regular diet, the high-fat diet significantly (*p* < 0.005) downregulated fatty acid synthase (FASN), growth factor signaling (phospho-IGFR, IGFBP2, phospho-Src, VEGFR2, etc.), and cell division/survival proteins (phospho-Rb, phospho-Wee1, phospho-p90RSK, phospho-NFκB, p-Rictor), but surprisingly activated P-AKT without the consequent downstream phosphorylation of GSK3. The high-fat diet also activated markers of autophagy (LC3A/B, WIPI1), cell cycle inhibition (p21, TIGAR), and cellular stress (Phospho-AMPK, Phospho-RPA32, P38-MAPK).

In mice fed with regular diet, IACS treatment induced complete stasis of A375R1 tumors and robust inhibition of UCSD354L tumor growth over 30 days of treatment (Fig. 6C and D). Over the same time course, STN treatment induced minimal inhibition of A375R1 and UCSD354L tumor growth. IACS + STN induced complete regression of A375R1 and stasis of UCSD354L tumors (Fig. 6C and D), suggesting potent tumor growth inhibition by the combination treatment. In mice fed with high-fat keto diet, A375R1 and UCSD354L tumor growth in vehicle-treated mice was significantly less (*p* < 0.001) than the growth observed in mice fed with regular diet (Fig. 6E and F). While this is consistent with ketogenic diet-induced ketosis in certain conditions [30, 31], one study showed that high-fat diet selectively promotes tumor growth of BRAF^*V600E*^-dependent human melanoma cells [29]. As our models are resistant to BRAFi, it is possible that they are not dependent on mutant-BRAF protein, which may potentially alter their response to high-fat keto diet. So, we tested the effect of high-fat keto diet on subcutaneous growth of parental A375 tumors that are dependent on BRAF^*V600E*^ and sensitive to BRAFi. Interestingly, the A375 tumors also showed significantly lower growth in mice fed with high-fat keto diet versus regular diet (Fig. S3B). In the high-fat keto diet–fed mice, STN treatment induced minimal inhibition of A375R1 and UCSD354L tumor growth compared with vehicle, as was also observed in mice fed with regular diet. IACS treatment however induced potent tumor regressions within 20 days, and IACS + STN completely eradicated the tumors of both models (Fig. 6E and F). Longer treatment times were not pursued as IACS and IACS + STN treatments induced > 15% weight loss within 25 days in mice fed with high-fat diet versus regular diet (Fig. S3C and S3D).

RPPA analysis of A375R1 tumor lysates from regular and high-fat keto diet–fed mice showed similar treatment-induced alterations compared with vehicle treatments (Fig. 6G and H). For example, IACS treatment activated phospho-AMPK and inhibited phospho-MAPK, while STN treatment inhibited IACS-induced phospho-AKT. The downstream alterations induced by these molecular effects were also similar between the regular and high-fat keto diet tumor samples. The higher potency of tumor growth inhibition in the high-fat keto diet cohorts could be a combination of ketogenesis and FA nutritional cutoff by the treatments. Compared with the *in vitro* cell line results, the tumor RPPA results additionally showed that IACS and IACS + STN inhibited the pro-tumorigenic hypoxia protein, HIF1α. Also, STN treatment upregulated PAR, and IACS counteracted it (Fig. 6G and H). These results suggest that IACS and STN cancel one another’s pro-tumorigenic protein signaling and enhance antitumor signaling.

## Discussion

Most BRAF^V600^-mutant melanoma patients treated with first-line standard of care, BRAF and MEK inhibitors, show impressive initial responses, but almost all experience disease relapse within a year [32]. While immune checkpoint inhibitors and T cell–mediated therapies offer long-term benefit [33], many patients do not respond to these treatments or eventually relapse after initial response [34, 35]. Novel second-line therapies are urgently needed to counteract refractory disease. Our combinatorial drug screen identified clinically relevant small molecules that showed impressive combination efficacy with IACS for inhibiting the growth of BRAFi/MEKi-resistant melanomas. Among these molecules that inhibit a variety of molecular targets, an HMGCRi, STN, showed the highest combination efficacy with IACS in validation studies. IACS combination with STN also induced cell death of BRAFi/MEKi-resistant melanomas.

For identifying the mechanistic basis of IACS + STN combination efficacy in these melanoma cells, we performed RPPA proteomics analysis, which revealed that IACS treatment inhibited MAPK signaling and FA synthesis, while activating AKT signaling. BRAFi/MEKi are well-known to activate AKT by feedback activation of RTK and other proteins in the PI3K pathway, which promote resistance [23]. HMGCRi, like STN, are known to inhibit AKT activity by inhibiting mevalonate pathway-induced isoprenylation of RAS, the upstream activator of AKT [36–38]. Indeed, combination of IACS with STN inhibited the IACS-induced AKT activation, resulting in the downregulation of cell cycle and survival proteins and activation of cell death proteins. As the scope of this study is limited to identifying the most potent combination treatment for improving the efficacy of IACS in BRAFi-resistant melanomas and testing their metabolic/molecular correlates of efficacy, we did not perform functional genetics analysis to tease out specific molecular players, for example PGC1α, as a potential causal factor of FA dependence in BRAFi-resistant melanomas.

**Fig. 7:**
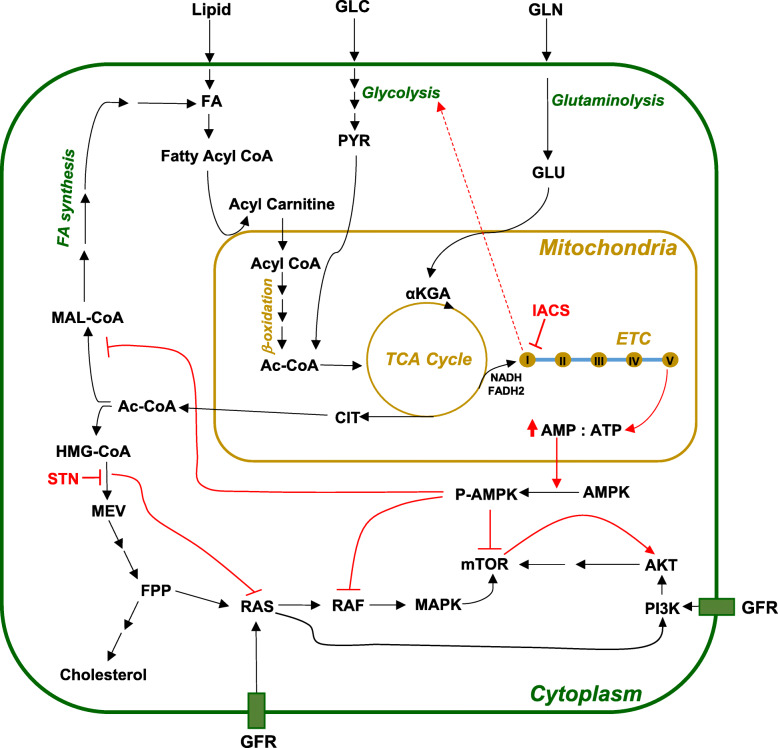
Mechanistic features of synergy between IACS and STN. IACS treatment inhibits mitochondrial complex I and ATP generation by the electron transport chain (ETC), resulting in activation of AMPK, inhibition of mTOR, MAPK, and FA synthesis. IACS treatment also decreases acetyl CoA (Ac-CoA) uptake into FA synthesis, but increases its uptake into HMGCoA synthesis and activates mevalonate (MEV) pathway. This in turn activates RAS-mediated AKT. STN inhibits HMGCR, resulting in the inhibition of MEV pathway and AKT. Combination of IACS with STN starves inhibits FA-dependent BRAF^V600E^ mutant melanoma cells by inhibition of FA and MEV pathways as well as MAPK and AKT pro-survival pathways

Bioenergetics flux analyses showed FA metabolic dependency in these BRAFi/MEKi-resistant melanomas, which suggested that FA metabolism is a potential therapeutic vulnerability if GLC (glucose) or GLN (glutamine) metabolism are stifled. The combined results of RPPA analysis and [^13^C]-GLC and [^13^C]-GLN tracing studies led us to conclude that IACS treatment inhibited FA synthesis, resulting in an accumulation of GLC- and GLN-derived acetyl CoA, which then fed the HMGCR-mediated mevalonate pathway and activated AKT. Conversely, STN treatment inhibited the mevalonate pathway, increasing the uptake of acetyl CoA for FA synthesis and inhibiting AKT activity (Fig. 7). These combined effects boost the antitumor activity of IACS + STN in FA metabolism-dependent melanomas.

The effects of ketogenic diet on mouse longevity and tumor growth were previously studied [39]. Our experiments with ketogenic diet were neither meant to replicate those studies nor to evaluate the effect of ketosis on tumor growth, but rather to specifically assess the effects of IACS and STN on tumor growth in a lipid-rich, GLC- and GLN-limiting nutritional context *in vivo*. With IACS and STN inhibiting the two critical branches of acetyl CoA–mediated lipid metabolism, nutritional deprivation of GLC and GLN in the ketogenic diet completely regressed the FA-dependent tumors, providing functional proof of the observed metabolic dependencies. Our RPPA results show that alteration of a balanced carbohydrate-rich diet towards a high-fat keto diet did not have any consequence on the molecular activities of IACS and STN, thus suggesting that inhibition of FA metabolism and mevalonate pathway is the functional basis of the tumor growth inhibition by the combination treatment. While these tumor growth inhibition results may support ketogenic dietary interventions for improving the efficacy of specific therapies in metabolically stratified tumors, our results also showed that IACS treatment led to extreme weight loss in the mice fed with ketogenic diet and hence should be interpreted with caution. Our observed effects with the high-fat keto diet may also be context-dependent, for example, the NSG mice used in our study may respond differently to this diet compared with nude mice used in another study which showed an increase of melanoma tumor growth in mice fed with high-fat diet [29]. As tumor metabolism is increasingly being implicated as a vital feature of cancer therapeutic response, it is important to explore such contextual differences in future studies.

Single-agent IACS treatment induced partial responses against hematological and solid cancers in early phase I clinical trials [16] (manuscript under preparation). Our previous study in mice models showed promising single-agent efficacy against BRAFi/MEKi-resistant melanomas, but unacceptable toxicity with MEKi combinations and insignificant benefit with BRAFi combinations [14]. Hence, there is a strong rationale to combine IACS with other clinically viable therapeutics that improve efficacy and maintain clinical tolerance. Based on our current study, it would be compelling for future clinical studies with IACS or other mitochondrial OxPhos targeting agents to specifically assess efficacy in patients who take HMGCRi for managing cardiovascular diseases. As the 1 mg/kg dose of STN in our study is at the higher end of the spectrum of HMGCRi use by patients with cardiovascular disease, dose relationships as well as differences in lipophilic and non-lipophilic HMGCRi could be explored in these studies prior to planning specific trials with OxPhos- and HMGCR-targeting agents.

Drugs that specifically target FA biosynthesis like SCD inhibitors could potentially be safer than ETC inhibitors like IACS. However, the pleiotropic effect of IACS (for example its inhibition of MAPK, AMPK, mTOR, FA) could be an important feature of its efficacy. STN is also known to exhibit pleiotropic effects as contributing factors for anticancer activity [40–42]. In addition to HMGCRi, other mevalonate pathway inhibitors may exhibit synergy with OxPhos- or FA-targeting agents and could be promising approaches against BRAFi/MEKi-resistant melanomas exhibiting FA dependency, which could be explored in future studies.

## Conclusions

Our study revealed an interesting dependency on FA metabolism in some BRAFi/MEKi-resistant melanomas, which drives a unique relationship between FA synthesis and HMGCR pathways. The significance of this relationship is emphasized by their positive association with MAPK and AKT pathways which are known to promote resistance to BRAFi/MEKi. This relationship is also a metabolic vulnerability that is responsive to combination therapy with IACS and STN. As safety profiles of STN and other HMGCRi are well established [43] and OxPhos-targeting agents are actively being pursued for treatment of therapy-resistant melanomas and other cancers, their combination is an important actionable therapeutic strategy against BRAFi/MEKi-resistant melanomas.

## Supplementary information

**Additional file 1.** Supplemental methods: combinatorial drug screen and stable isotope tracing analysis of ^13^C_6_-glucose and ^13^C_5_-glutamine.

**Additional file 2: Figure S1.** (**A-B**) A375R1 cells were seeded in 96 well plates (10^3^ cells/well) and treated with GSK (*A*) or BKM120 (*B*) and their combinations with IACS at 1:1 concentration shown on the x-axes. Cell growth inhibition was determined after 72 h using Cell Titer Blue reagent. (**C**) The same experiment as above was performed on normal epidermal melanocytes with the indicated treatments, and cell growth inhibition was determined after 72 h using Cell Titer Blue reagent. (**D**) The same experiment as above was performed in A375R1 cells with the indicated inhibitors, but in this case, cell growth inhibition was determined after 72 h using Crystal Violet dye staining. In panels *A-D*, data is normalized to vehicle-treated cells and is average of triplicates, with error bars representing *SD*, and colored asterisks representing significant differences (*=<0.033; **=<0.002; ***=<0.001) in effects for combination treatment versus individual probes (red) or IACS (black). (**E** and **F**) A375R1 and UCSD354L cells were treated with 100 nM Dabrafenib (DAB) or its combination with IACS (100 nM) or STN (1 μM) for 72 h and cell cycle profiles were generated using propidium iodide-FACS analysis, which included sub-G1 (dead cell) population. Data are plotted as bar graphs of triplicates; error bars represent *SD*; Asterisk (*) represents significant differences (*p*<0.05) of DAB+STN compared to the other treatments shown. (**G-J**) Western blot bands from P-AKT_Thr308 and P-AMPK_Thr172 protein staining were quantified using Image J software and represented as bars graphs of quantified area in square pixels for each of the protein bands (y-axis) versus treatments (x-axis) for A375R1 (*G and H*) and UCSD354L (*I and J*) cells. **Figure S2. (A, B)** The Seahorse fuel-flex assay was performed on MEL624 *(A)* and WM1799 *(B)* cells to determine their dependency (blue) on glucose (GLC), glutamine (GLN) and fatty acids (FA), and their flexibility (orange) to utilize either of the single nutrients when the other two are inhibited. Data is quadruplicates; error bars, SD. **(C, D)** MEL624 *(C)* and WM1799 *(D)* cells were seeded in 96 well plates (10^3^ cells/well) and treated with IACS or STN or their combinations at 1:1 concentration shown on the x-axes. Cell growth inhibition was determined after 72 h using Cell Titer blue reagent. Data is normalized to vehicle-treated cells and is average of triplicates, with error bars representing *SD*. **(E)** A375R1 cells were seeded in 96 well plates (10^3^ cells/well) and treated with ETMR or STN at the concentrations shown on the x-axes. Cell growth inhibition was determined after 72 h using Cell Titer blue reagent. Data is normalized to vehicle-treated cells and is average of triplicates, with error bars representing *SD*. (**F**) A375R1 cells (1.5x10^4^) seeded in 96 well plates were subjected to the same consecutive inhibitor treatments and incubation conditions as in Fig. 4D. The cells were then detached by trypsinization, live cells were counted using trypan blue dye exclusion and the results plotted as bar graphs of viable cells following each subsequent treatment. Data is triplicates; error bars, *SD*. (**G**) Relative abundance of intracellular acetyl CoA (Ac-CoA) in A375R1 cells treated with vehicle (V), IACS (I), STN (S) or their combination (I+S) for 12 hours. (**H, I**) Fractional labeling of acetoacetate by [U-^13^C]-GLC (glucose) *(H)* or [U-^13^C]-GLN (glutamine) *(I)* following treatment with IACS (I) and STN (S) or their combination (I+S) for 12 h. **Figure S3.** (**A**) Heatmap showing unsupervised clustering analysis of significantly (*p*<0.005) altered proteins in UCSD354L tumors harvested from mice fed with regular (Reg) or high-fat keto diet and analyzed by RPPA. The heatmap shows Pearson correlation of significantly (*p*<0.005) different proteins in tumors from mice fed with high fat keto (Hi-fat) versus regular (Reg) diet. Intensity ranges of lowest (blue) and highest (red) protein levels are indicated at the bottom of the heatmap. (**B**) Sub-cutaneous tumor growth of parental A375 cells in mice fed with Regular diet or high-fat keto diet. (**C** and **D**) Percent changes in body weights of A375R1 tumor bearing mice fed with regular (Reg) diet (*C*) or high-fat keto diet (Hi-fat) (*D*), and treated with Vehicle, 5mg/kg IACS, 1mg/kg STN or their combinations over the number of days shown. Mice weight data is from eight mice/group, error bars, standard error of mean (*SEM*).

**Additional file 3: Table S1.** Inhibitors (probes) in the combinatorial drug screen, their molecular targets and cellular pathways targeted. **Table S2.** IC50 values of single agent inhibitors (probes) and their combinations with IACS-010759 (IACS), derived from growth inhibition curve analysis using GraphPad Prism.

## Data Availability

The datasets used and/or analyzed during the current study are available from the corresponding author on reasonable request.

## Abbreviations

BRAFi: BRAF inhibitors
OxPhos: Oxidative phosphorylation
IACS: IACS-010759
HMGCRi: Hydroxymethylglutaryl-coenzyme A reductase inhibitors
MAPK: Mitogen-activated protein kinase
HMGCR: Hydroxymethylglutaryl-coenzyme A reductase
DMSO: Dimethyl sulfoxide
STN: Atorvastatin
DAPI: 4′,6-diamidino-2-phenylindole
OCR: Oxygen consumption rate
ECAR: Extracellular acidification rate
MDACC: MD Anderson Cancer Center
RPPA: Reverse-Phase Protein Array
NSG: NOD scid gamma
DAB: Dabrafenib
AMPK: AMP-activated protein kinase
AKT: Protein kinase B
GLC: Glucose
GLN: Glutamine
FA: Fatty acids
ETMR: Etomoxir, 2[6(4-chlorophenoxy)hexyl]oxirane-2-carboxylate
LC-MS: Liquid chromatography–mass spectrometry
G6P: Glucose 6 phosphate
PEP: Phosphoenolpyruvate
PYR: Pyruvate
TCA cycle: Tricarboxylic acid cycle
CIT: Citrate
αKGA: α-Ketoglutarate
SUC: Succinate
Ac-CoA: Acetyl CoA
AcAc: Acetoacetate
MEV: Mevalonate
MAL-CoA: Malonyl CoA
LIPO: Lipoic acid
FASN: Fatty acid synthase
ETC: Electron transport chain
