# Supplementary material for: Combined inhibition of HMGCoA reductase and mitochondrial complex I induces tumor regression of BRAF inhibitor-resistant melanomas
Source: Cancer Metab. 2022 Feb 22;10:6. doi: 10.1186/s40170-022-00281-0 (PMC8862475; doi:10.1186/s40170-022-00281-0)
Supplement: Supplementary file 1 — Additional file 1. Supplemental methods: combinatorial drug screen and stable isotope tracing analysis of 13C6-glucose and 13C5-glutamine. [file 40170_2022_281_MOESM1_ESM.docx]

**Supplemental Methods**

***Combinatorial drug screen:***

This was performed at the High Throughput Screening Center at the Institute of Bioscience and Technology, Texas A&M University, to evaluate the combination efficacy of IACS-010759 with 320 clinically relevant compounds with mechanistically diverse targets.

*Cell Culture:* Cell lines underwent a systematic growth analysis to determine their rate of growth. Results from the growth assays were used to identify cell-seeding densities that maintain log-phase growth and result in approximately 70-80% confluence at the conclusion of a 96-hour assay window. Cells maintained in tissue culture flasks were trypsinized and re-suspended in 10mL of serum-containing media and counted using a TC10 (Bio-Rad) automated cell counter. The cell-containing media was then diluted to 2000 cells/ml from which a 2-fold serial dilution is performed. The cells were then seeded in a 384-well plate (50 µL/well and 8 wells per dilution). The plated cells were allowed to attach at room temperature for 45-60 min and then moved into a humidified (≥ 95% RH) cell culture incubator at 37 ^o^C and 5% CO_2_ overnight. At 24, 48, 72, 96, 120 and 168 h after plating, 8 replicate wells from each cell dilution were stained with 10ug/mL Hoescht 33342 and counted. The doubling times were calculated using the formula: Td = 3*(LOG (2)/LOG (cell number at time after plating/cell number at 24 h)).

*High throughput screening combination of Anchor and Probes:* For combination assays, 2000 cells/well suspended in 50µl media were transferred by a Multidrop Combi liquid dispenser (ThermoFisher Scientific) into 384-well black polystyrene, clear F-bottom plates (Greiner 781091). All plates were kept at room temperature for 45-60min immediately after plating and then moved into a Cytomat 6000A *ThermoFisher Scientific) humidified, 5% CO2 cell culture incubator integrated into a Labcyte Access platform overnight in single racked position. Plates were moved from the incubator on the Access platform to the Labcyte Echo 550 acoustic dispenser to dispense 5nl and 50nl from appropriate 1000x stock concentrations of probes and anchor (prepared in 100% DMSO). A total of 320 probe compounds (selected from Selleck Bioactive collection on the basis of clinical and mechanistic relevance to the study) were diluted to 1 mM stocks in DMSO. Each probe was tested in four concentrations: 1, 0.1, 0.01, and 0.001 µM in a pairwise combination with four concentrations of the anchor, IACS-010759 (IACS). The final concentration of DMSO did not exceed 0.2% (v/v). Each assay plate contained 8 pairs of anchor-probe combinations which are run in duplicate on the same plate. Each plate contains a vehicle treated negative control, a 10 µM Doxorubicin positive control, and two 8 point dose responses gradient (starting at 10 µM) of doxorubicin and IACS. A non-treated plate was fixed and nuclei DAPI stained at the time of drug addition (Day 0) to provide the number of cells present per well at the time of treatment. After cells and drugs incubated for 72hr, they were fixed with 0.4% Paraformaldehyde, washed and stained with DAPI using Tecan Hydrospeed plate washer and Multidrop Combi Dispenser. Plates were imaged on an IN Cell Analyzer 6000 confocal imaging platform (GE Health Care) and nuclei counted using the analysis program from Developer software (GE Health Care).

*Rigor and reproducibility analysis:* A running statistical analysis was performed in accordance with the NCATS Assay Guidance Manual to demonstrate reproducibility and robustness of combination screening assays ((Iversen et al., 2004b). (Iversen et al., 2004a) To monitor assay robustness, the robust Z’-factor (which uses median and MAD instead of mean and standard deviation) was calculated from on-plate DMSO and 10 uM doxorubicin control wells. For this statistic, values over 0.5 are considered to represent a highly robust assay. We observed a mean robust z’-factor of 0.85 and 0.83 for A375R1 and UCSD354L cell lines, respectively. In order to monitor assay reproducibility, we calculate the minimum significant ratio (MSR) from the IC50 of an on-plate doxorubicin dose response curve. In general, a MSR of less than three is considered to be a highly reproducible assay. We observed MSR values of 1.74 and 2.24 for A375R1 and UCSD354 cell lines respectively. Collectively, these statistical analyses show a highly robust and reproducible cytoxicity assay with minimal to no drift.

*Statistical analysis of combinations:* A bootstrapped bliss independence model was used to calculate drug synergy. This model assumes that if the observed activity of two independently acting drugs is in excess of the additive product of the individual components, then the drugs are acting synergistically. The bliss model requires raw data to first be normalized as percent inhibition, which we calculated by normalizing each data point to the on-plate negative control so that a value of 0 (zero) represents no compound effect and a value of 1 represents a full kill with no cells remaining in the well. To further minimize the false positive rate and increase the rigor of the standard bliss independence model, we adapted the methods described in detail by Zhao et al.(Zhao et al., 2014). In this implementation, a theoretical bliss independence surface is fit across all combination of concentrations using on-plate single agent dose response curves. The predicted bliss surface is then compared to the empirically observed value to identify synergistic domains. The confidence interval around any combination is then estimated using a bootstrapping method, where the predicted and observed response at any given concentration are synthetically expanded using a random normal distribution and compared over 1000 iterations. Thus providing an outlier robust method to determine the confidence in combinations with relatively few replicates. As an objective cut-off for synergy, we use a 15% difference between the lower limit (5th percentile) of observed wells to the predicted bliss surface.

***Stable isotope tracing analysis of*** ***^13^C_6_-glucose and ^13^C_5_-glutamine*.**

This was performed at the MDACC Metabolomics Core Facility for tracing of [^13^C] from glucose or glutamine into downstream metabolites in the glycolysis, TCA cycle, fatty acid synthesis, and mevalonate pathways.

*Cell treatment and Metabolite Extraction:* Melanoma cells (3x10^6^ cells/150mm dish) in RPMI media containing 5% fetal bovine serum were seeded overnight. On the day of the experiment, the media was removed, cells were washed once in PBS and replenished with pyruvate-free media containing dialyzed serum and 10 mM [U-^13^C]-glucose or 2 mM [U-^13^C]-glutamine. After one hour, cells were then treated with the indicated inhibitors for 12 h.

Metabolites were extracted in 2 mL of ice-cold 40/40/20 acetonitrile/methanol/water mixture after scraping them into solution. The samples were vortexed and centrifuged at 17,000 *g* for 5 min at 4°C, and supernatants were transferred to clean tubes and evaporated to dryness under nitrogen. Cell pellets were saved for DNA extraction. Metabolite lysates were subjected to LC-MS and IC-MS analysis as described below. DNA was extracted from the cell pellets using Qiagen RNeasy kit and quantified in a Nanodrop spectrophotometer.

*Targeted HRAM-LC/MS analysis of CoA intermediates.*

For CoA analysis, samples were reconstituted in 100 µL 5 mM ammonium acetate in water, then 15 µL was injected for analysis by liquid chromatography (LC)-MS. LC mobile phase A (MPA; weak) was 5 mM ammonium acetate in water, and mobile phase B (MPB; strong) was methanol. A Thermo Vanquish LC system included an Accucore C30 column (2.6 µm particle size, 150 x 2.1 mm) with column compartment kept at 30°C. The autosampler tray was chilled to 4°C. The mobile phase flow rate was 300 µL/min, and the gradient elution program was: 0-2 min, 5% MPB; 2-4 min, 5-10% MPB; 4-6.5 min, 10-95% MPB; 6.5-14.5 min, 95% MPB; 14.5-15 min, 95-5% MPB; 15-20 min, 5% MPB. The total run time was 20 min. Data were acquired using a Thermo Orbitrap Fusion Tribrid Mass Spectrometer under ESI positive ionization mode at a resolution of 240,000.

*Targeted HRAM-IC/MS analysis of glycolysis and TCA cycle intermediates and Lipoic Acid:*

Targeted HRAM-IC/MS analysis was performed using a Dionex ICS-5000+ capillary ion chromatography (IC) system coupled to an OrbiTrap Fusion Mass Spectrometer using IonPac AG-11-HC guard and IonPac AS11-HC analytical columns (all instrumental components and columns were from Thermo Fisher Scientific). The IC method used deionized water (18.2 MΩ-cm) as the input solvent, a column temperature of 30°C, autosampler tray chilled to 10°C, a mobile phase flow rate of 0.36 mL/min, and a gradient elution program specified as follows: 0 – 25 min, 1 mM – 35 mM KOH; 25 – 39 min, 35 – 99 mM KOH; 39 – 49 min, 99 mM KOH; 49 – 50 min, 99 – 1 mM KOH. KOH was produced in the chromatographic flow path by an in situ Eluent Generator and removed by a suppressor prior to introduction to the MS system. The total run time was 50 min. To improve desolvation in the ionization source for better sensitivity, methanol was delivered by an external pump and combined with the eluent via a low dead-volume mixing tee. The H-ESI ionization source and Global MS parameters for the acquisition parameters for the HRAM-IC/MS method were specified as follows: scan range: m/z: 80-800; multiplex ions: false; isolation mode: quadrupole; detector type: Orbitrap; Orbitrap resolution: 240,000 (at m/z 200); RF lens (%): 50; AGC Target: 200,000; injection ions for all available parallelizable time: true; maximum injection time (ms): 100; microscans: 1; data type: profile; polarity: negative; source fragmentation: disabled; use EASY-IC: true; include start and end times: false; scan cycle time: 3 seconds. The raw data files were imported to Thermo Trace Finder software for final analysis and determination of relative abundances of the metabolites in the samples. These were normalized against cellular DNA levels as described (Silva PL et al., Anal Chem.2013, PMID:24011029) to adjust for cell number variability in cells harvested at the end of the experiments. ElemCor software (Du D et al., BMC Bioinformatics 20, 89; 2019. PMID:30782135) was used for natural abundance correction and enrichment calculation for ^13^C isotopolog analysis. An enrichment score for each metabolite was generated by adding the istopolog peak areas. DNA-normalized ^13^C enrichment of the metabolites were represented in bar graphs of fractional abundances, with indicated treatments shown on the X-axis.
