# Supplementary material for: Combined inhibition of HMGCoA reductase and mitochondrial complex I induces tumor regression of BRAF inhibitor-resistant melanomas
Source: Cancer Metab. 2022 Feb 22;10:6. doi: 10.1186/s40170-022-00281-0 (PMC8862475; doi:10.1186/s40170-022-00281-0)
Supplement: Supplementary file 2 — Additional file 2: Figure S1. (A-B) A375R1 cells were seeded in 96 well plates (103 cells/well) and treated with GSK (A) or BKM120 (B) and their combinations with IACS at 1:1 concentration shown on the x-axes. Cell growth inhibition was determined after 72 h using Cell Titer Blue reagent. (C) The same experiment as above was performed on normal epidermal melanocytes with the indicated treatments, and cell growth inhibition was determined after 72 h using Cell Titer Blue reagent. (D) The same experiment as above was performed in A375R1 cells with the indicated inhibitors, but in this case, cell growth inhibition was determined after 72 h using Crystal Violet dye staining. In panels A-D, data is normalized to vehicle-treated cells and is average of triplicates, with error bars representing SD, and colored asterisks representing significant differences (*=<0.033; **=<0.002; ***=<0.001) in effects for combination treatment versus individual probes (red) or IACS (black). (E and F) A375R1 and UCSD354L cells were treated with 100 nM Dabrafenib (DAB) or its combination with IACS (100 nM) or STN (1 μM) for 72 h and cell cycle profiles were generated using propidium iodide-FACS analysis, which included sub-G1 (dead cell) population. Data are plotted as bar graphs of triplicates; error bars represent SD; Asterisk (*) represents significant differences (p<0.05) of DAB+STN compared to the other treatments shown. (G-J) Western blot bands from P-AKT_Thr308 and P-AMPK_Thr172 protein staining were quantified using Image J software and represented as bars graphs of quantified area in square pixels for each of the protein bands (y-axis) versus treatments (x-axis) for A375R1 (G and H) and UCSD354L (I and J) cells. Figure S2. (A, B) The Seahorse fuel-flex assay was performed on MEL624 (A) and WM1799 (B) cells to determine their dependency (blue) on glucose (GLC), glutamine (GLN) and fatty acids (FA), and their flexibility (orange) to utilize either of the single nutrients when the other [file 40170_2022_281_MOESM2_ESM.pptx]

## Slide 1
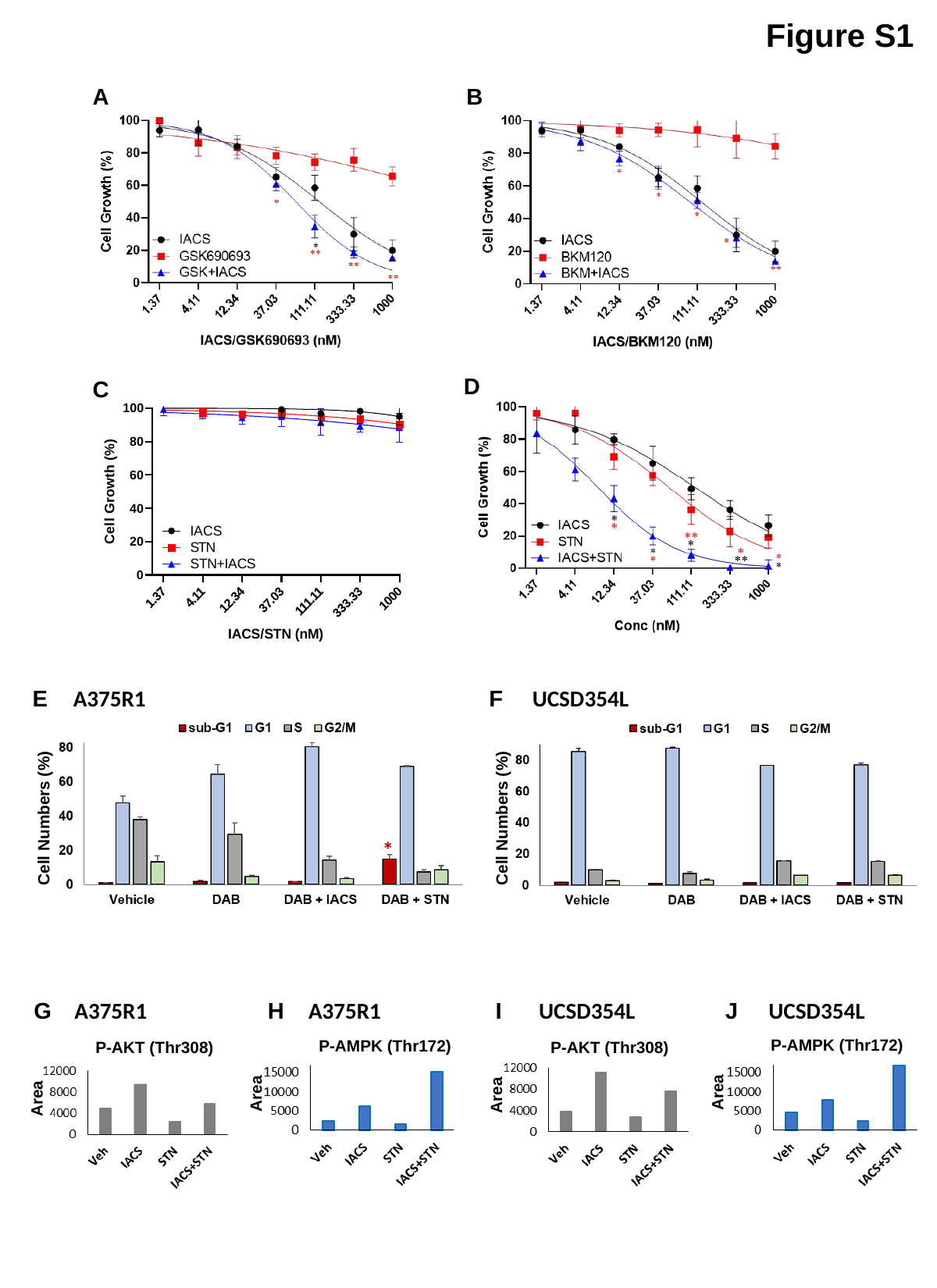

Figure S1
B
A
D
C
UCSD354L
A375R1
E
F
Cell Numbers (%)
Cell Numbers (%)
*
A375R1
A375R1
UCSD354L
UCSD354L
G
H
I
J
P-AMPK (Thr172)
P-AMPK (Thr172)
P-AKT (Thr308)
P-AKT (Thr308)
Area
Area
Area
Area

## Slide 2
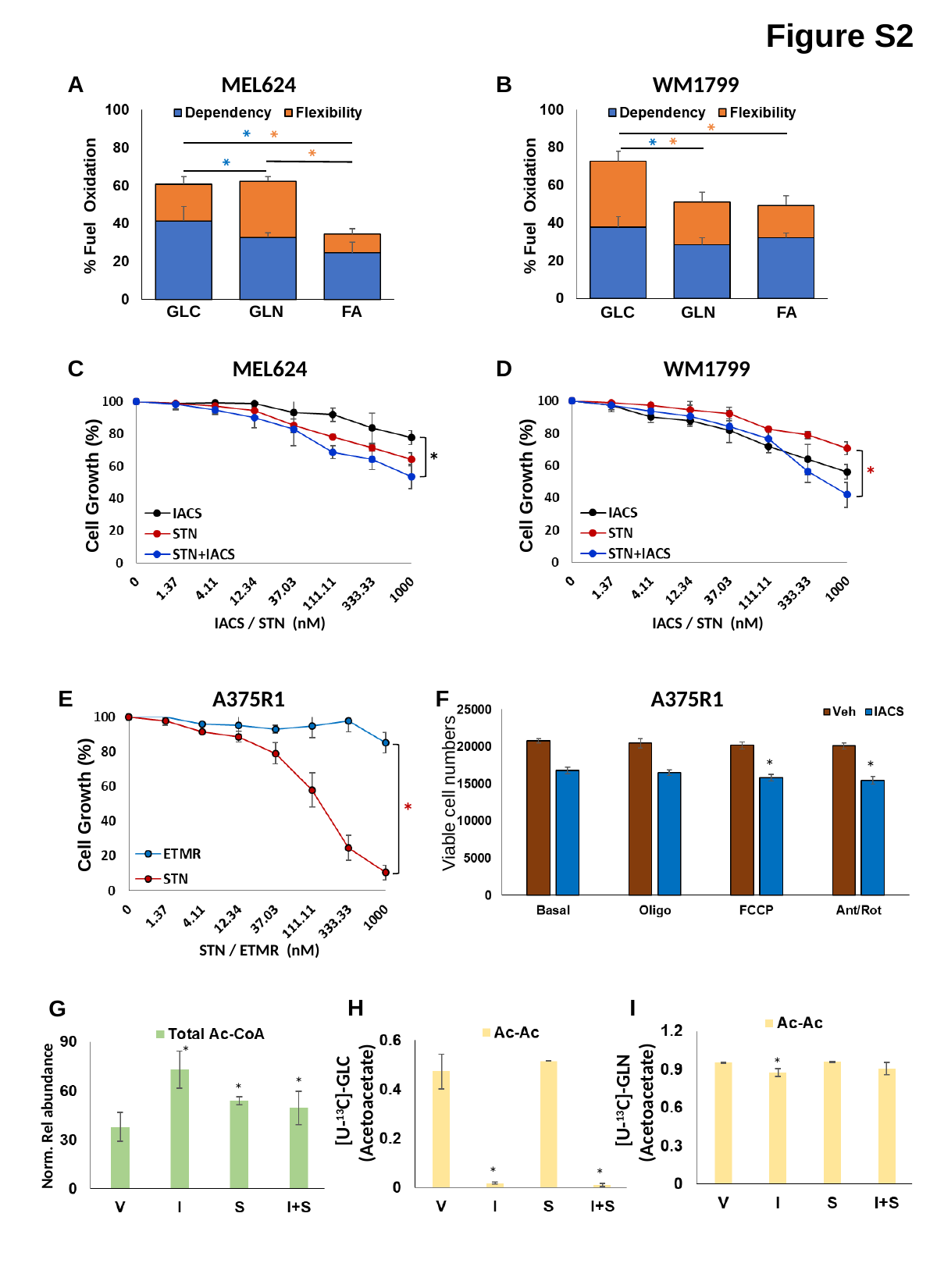

Figure S2
A
B
WM1799
MEL624
*
*
*
*
*
*
*
% Fuel Oxidation
% Fuel Oxidation
GLN
FA
GLC
GLN
FA
GLC
D
WM1799
C
MEL624
*
*
Cell Growth (%)
Cell Growth (%)
IACS / STN (nM)
IACS / STN (nM)
E
A375R1
A375R1
F
*
*
Viable cell numbers
Cell Growth (%)
*
STN / ETMR (nM)
I
H
G
*
*
*
*
 [U-13C]-GLN
(Acetoacetate)
 [U-13C]-GLC
(Acetoacetate)
Norm. Rel abundance
*
*

## Slide 3
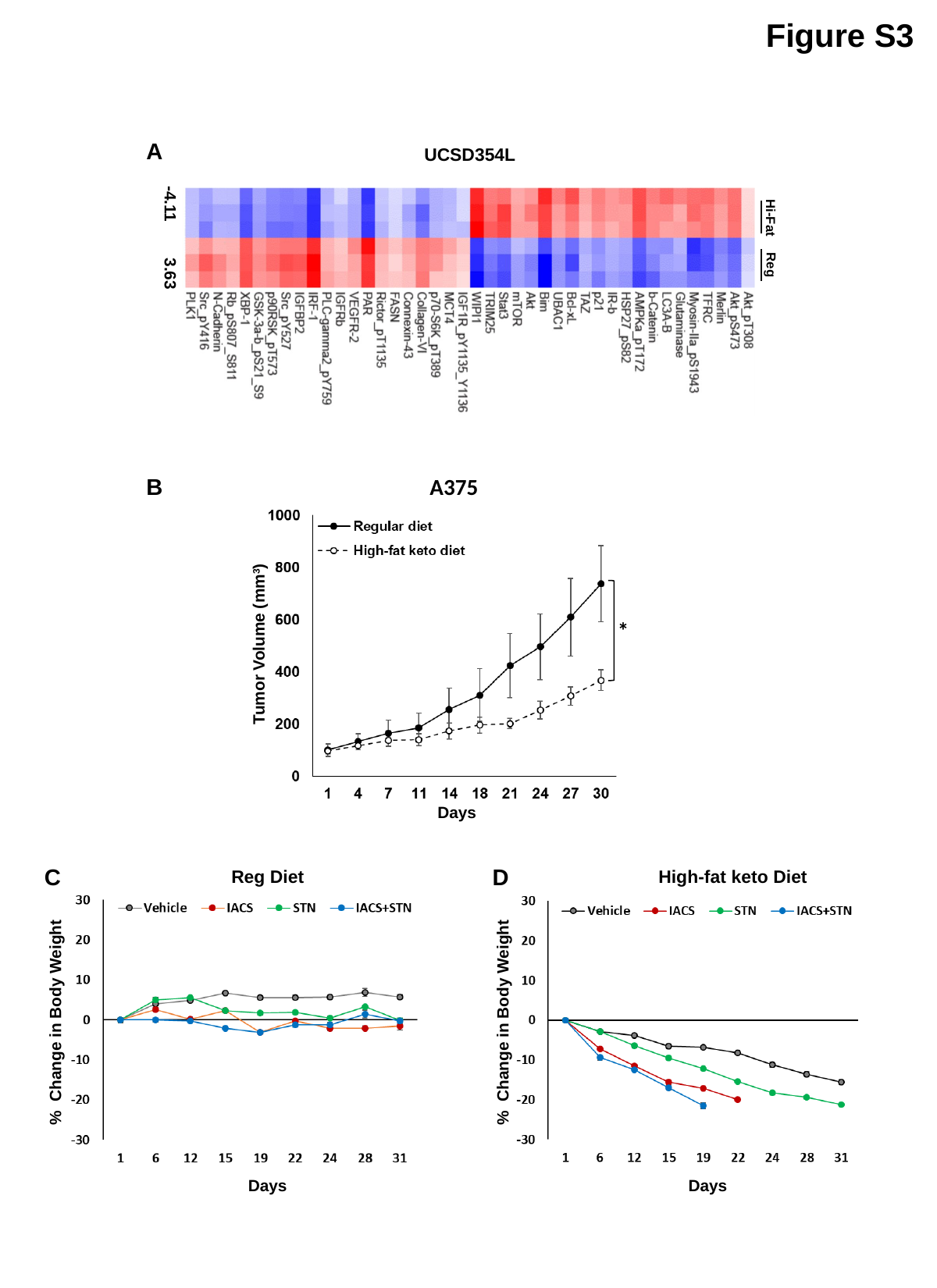

Figure S3
A
UCSD354L
Hi-Fat
-4.11 3.63
Reg
B
A375
*
Tumor Volume (mm3)
Days
C
D
Reg Diet
High-fat keto Diet
% Change in Body Weight
% Change in Body Weight
Days
Days
