# Supplementary material for: Combined inhibition of HMGCoA reductase and mitochondrial complex I induces tumor regression of BRAF inhibitor-resistant melanomas
Source: Cancer Metab. 2022 Feb 22;10:6. doi: 10.1186/s40170-022-00281-0 (PMC8862475; doi:10.1186/s40170-022-00281-0)
Supplement: Supplementary file 3 — Additional file 3: Table S1. Inhibitors (probes) in the combinatorial drug screen, their molecular targets and cellular pathways targeted. Table S2. IC50 values of single agent inhibitors (probes) and their combinations with IACS-010759 (IACS), derived from growth inhibition curve analysis using GraphPad Prism. [file 40170_2022_281_MOESM3_ESM.pptx]

## Slide 1
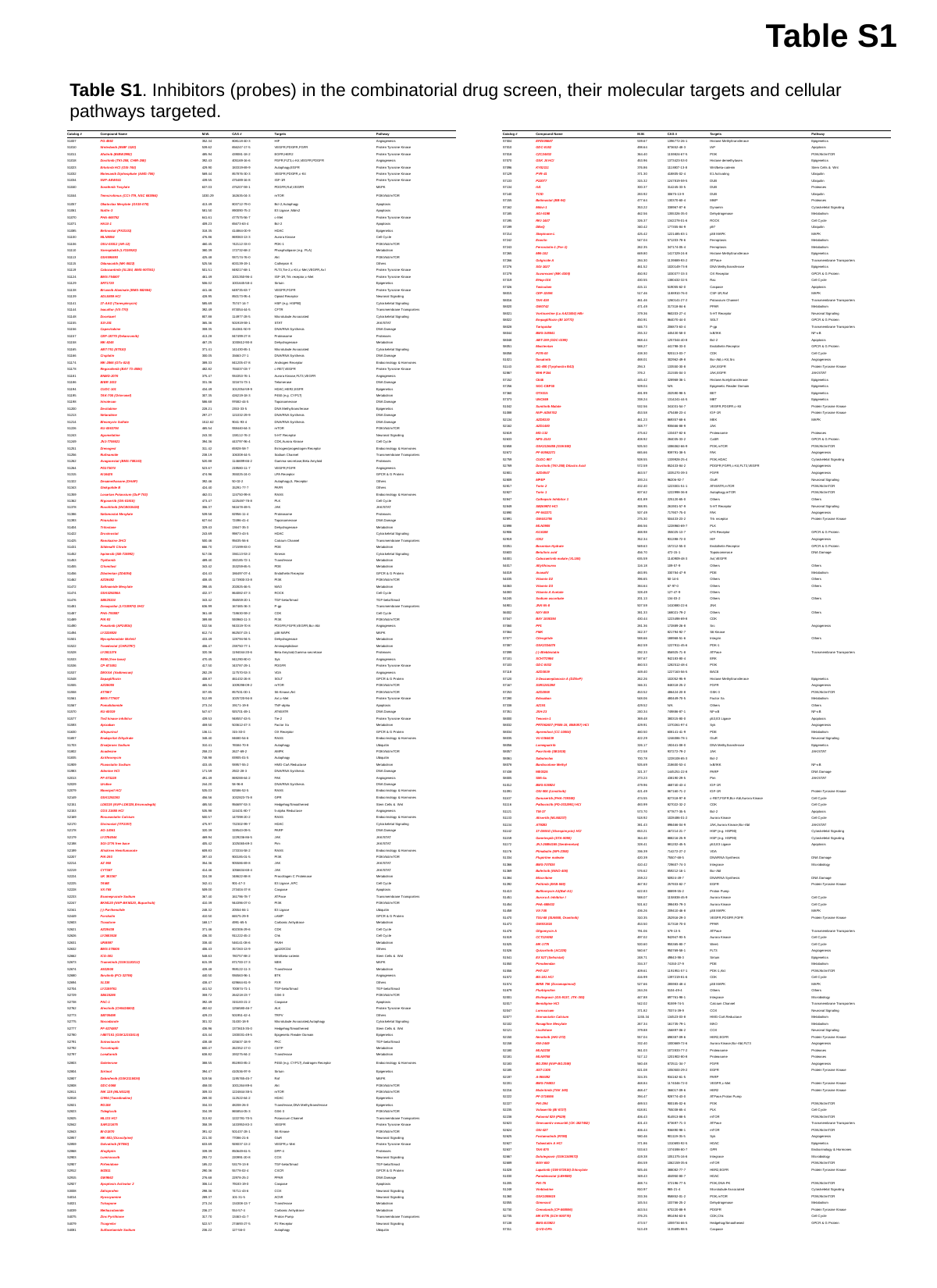

Table S1
Table S1. Inhibitors (probes) in the combinatorial drug screen, their molecular targets and cellular pathways targeted.
| Catalog # | Compound Name | M.W. | CAS # | Targets | Pathway |
| --- | --- | --- | --- | --- | --- |
| S1007 | FG-4592 | 352.34 | 808118-40-3 | HIF | Angiogenesis |
| S1010 | Nintedanib (BIBF 1120) | 539.62 | 656247-17-5 | VEGFR,PDGFR,FGFR | Protein Tyrosine Kinase |
| S1011 | Afatinib (BIBW2992) | 485.94 | 439081-18-2 | EGFR,HER2 | Protein Tyrosine Kinase |
| S1018 | Dovitinib (TKI-258, CHIR-258) | 392.43 | 405169-16-6 | FGFR,FLT3,c-Kit,VEGFR,PDGFR | Angiogenesis |
| S1023 | Erlotinib HCl (OSI-744) | 429.90 | 183319-69-9 | Autophagy,EGFR | Protein Tyrosine Kinase |
| S1032 | Motesanib Diphosphate (AMG-706) | 569.44 | 857876-30-3 | VEGFR,PDGFR,c-Kit | Protein Tyrosine Kinase |
| S1034 | NVP-AEW541 | 439.55 | 475489-16-8 | IGF-1R | Protein Tyrosine Kinase |
| S1040 | Sorafenib Tosylate | 637.03 | 475207-59-1 | PDGFR,Raf,VEGFR | MAPK |
| S1044 | Temsirolimus (CCI-779, NSC 683864) | 1030.29 | 162635-04-3 | mTOR | PI3K/Akt/mTOR |
| S1057 | Obatoclax Mesylate (GX15-070) | 413.49 | 803712-79-0 | Bcl-2,Autophagy | Apoptosis |
| S1061 | Nutlin-3 | 581.50 | 890090-75-2 | E3 Ligase ,Mdm2 | Apoptosis |
| S1070 | PHA-665752 | 641.61 | 477575-56-7 | c-Met | Protein Tyrosine Kinase |
| S1071 | HA14-1 | 409.23 | 65673-63-4 | Bcl-2 | Apoptosis |
| S1085 | Belinostat (PXD101) | 318.35 | 414864-00-9 | HDAC | Epigenetics |
| S1100 | MLN8054 | 476.86 | 869363-13-3 | Aurora Kinase | Cell Cycle |
| S1106 | OSU-03012 (AR-12) | 460.45 | 742112-33-0 | PDK-1 | PI3K/Akt/mTOR |
| S1110 | Varespladib (LY315920) | 380.39 | 172732-68-2 | Phospholipase (e.g. PLA) | Metabolism |
| S1113 | GSK690693 | 425.48 | 937174-76-0 | Akt | PI3K/Akt/mTOR |
| S1115 | Odanacatib (MK-0822) | 525.56 | 603139-19-1 | Cathepsin K | Others |
| S1119 | Cabozantinib (XL184, BMS-907351) | 501.51 | 849217-68-1 | FLT3,Tie-2,c-Kit,c-Met,VEGFR,Axl | Protein Tyrosine Kinase |
| S1124 | BMS-754807 | 461.49 | 1001350-96-4 | IGF-1R,Trk receptor,c-Met | Protein Tyrosine Kinase |
| S1129 | SRT1720 | 506.02 | 1001645-58-4 | Sirtuin | Epigenetics |
| S1138 | Brivanib Alaninate (BMS-582664) | 441.46 | 649735-63-7 | VEGFR,FGFR | Protein Tyrosine Kinase |
| S1139 | ADL5859 HCl | 428.95 | 850173-95-4 | Opioid Receptor | Neuronal Signaling |
| S1141 | 17-AAG (Tanespimycin) | 585.69 | 75747-14-7 | HSP (e.g. HSP90) | Cytoskeletal Signaling |
| S1144 | Ivacaftor (VX-770) | 392.49 | 873054-44-5 | CFTR | Transmembrane Transporters |
| S1148 | Docetaxel | 807.88 | 114977-28-5 | Microtubule Associated | Cytoskeletal Signaling |
| S1155 | S3I-201 | 365.36 | 501919-59-1 | STAT | JAK/STAT |
| S1156 | Capecitabine | 359.35 | 154361-50-9 | DNA/RNA Synthesis | DNA Damage |
| S1157 | CEP-18770 (Delanzomib) | 413.28 | 847499-27-8 | Proteasome | Proteases |
| S1158 | MK-8245 | 467.25 | 1030612-90-8 | Dehydrogenase | Metabolism |
| S1165 | ABT-751 (E7010) | 371.41 | 141430-65-1 | Microtubule Associated | Cytoskeletal Signaling |
| S1166 | Cisplatin | 300.05 | 15663-27-1 | DNA/RNA Synthesis | DNA Damage |
| S1174 | MK-2866 (GTx-024) | 389.33 | 841205-47-8 | Androgen Receptor | Endocrinology & Hormones |
| S1178 | Regorafenib (BAY 73-4506) | 482.82 | 755037-03-7 | c-RET,VEGFR | Protein Tyrosine Kinase |
| S1181 | ENMD-2076 | 375.47 | 934353-76-1 | Aurora Kinase,FLT3,VEGFR | Angiogenesis |
| S1186 | BIBR 1532 | 331.36 | 321674-73-1 | Telomerase | DNA Damage |
| S1194 | CUDC-101 | 434.49 | 1012054-59-9 | HDAC,HER2,EGFR | Epigenetics |
| S1195 | TAK-700 (Orteronel) | 307.35 | 426219-18-3 | P450 (e.g. CYP17) | Metabolism |
| S1198 | Irinotecan | 586.68 | 97682-44-5 | Topoisomerase | DNA Damage |
| S1200 | Decitabine | 228.21 | 2353-33-5 | DNA Methyltransferase | Epigenetics |
| S1213 | Nelarabine | 297.27 | 121032-29-9 | DNA/RNA Synthesis | DNA Damage |
| S1214 | Bleomycin Sulfate | 1512.62 | 9041-93-4 | DNA/RNA Synthesis | DNA Damage |
| S1226 | KU-0063794 | 465.54 | 938440-64-3 | mTOR | PI3K/Akt/mTOR |
| S1243 | Agomelatine | 243.30 | 138112-76-2 | 5-HT Receptor | Neuronal Signaling |
| S1249 | JNJ-7706621 | 394.36 | 443797-96-4 | CDK,Aurora Kinase | Cell Cycle |
| S1251 | Dienogest | 311.42 | 65928-58-7 | Estrogen/progestogen Receptor | Endocrinology & Hormones |
| S1256 | Rufinamide | 238.19 | 106308-44-5 | Sodium Channel | Transmembrane Transporters |
| S1262 | Avagacestat (BMS-708163) | 520.88 | 1146699-66-2 | Gamma-secretase,Beta Amyloid | Proteases |
| S1264 | PD173074 | 523.67 | 219580-11-7 | VEGFR,FGFR | Angiogenesis |
| S1315 | Ki16425 | 474.96 | 355025-24-0 | LPA Receptor | GPCR & G Protein |
| S1322 | Dexamethasone (DHAP) | 392.46 | 50-02-2 | Autophagy,IL Receptor | Others |
| S1343 | Ginkgolide B | 424.40 | 15291-77-7 | PAFR | Others |
| S1359 | Losartan Potassium (DuP 753) | 462.01 | 124750-99-8 | RAAS | Endocrinology & Hormones |
| S1362 | Rigosertib (ON-01910) | 473.47 | 1225497-78-8 | PLK | Cell Cycle |
| S1378 | Ruxolitinib (INCB018424) | 306.37 | 941678-49-5 | JAK | JAK/STAT |
| S1386 | Nafamostat Mesylate | 539.58 | 82956-11-4 | Proteasome | Proteases |
| S1393 | Pirarubicin | 627.64 | 72496-41-4 | Topoisomerase | DNA Damage |
| S1404 | Trilostane | 329.43 | 13647-35-3 | Dehydrogenase | Metabolism |
| S1422 | Droxinostat | 243.69 | 99873-43-5 | HDAC | Cytoskeletal Signaling |
| S1425 | Ranolazine 2HCl | 500.46 | 95635-56-6 | Calcium Channel | Transmembrane Transporters |
| S1431 | Sildenafil Citrate | 666.70 | 171599-83-0 | PDE | Metabolism |
| S1452 | Ispinesib (SB-715992) | 517.06 | 336113-53-2 | Kinesin | Cytoskeletal Signaling |
| S1453 | Tipifarnib | 489.40 | 192185-72-1 | Transferase | Metabolism |
| S1455 | Cilomilast | 343.42 | 153259-65-5 | PDE | Metabolism |
| S1456 | Zibotentan (ZD4054) | 424.43 | 186497-07-4 | Endothelin Receptor | GPCR & G Protein |
| S1462 | AZD6482 | 408.45 | 1173900-33-8 | PI3K | PI3K/Akt/mTOR |
| S1472 | Safinamide Mesylate | 398.45 | 202825-46-5 | MAO | Metabolism |
| S1474 | GSK429286A | 432.37 | 864082-47-3 | ROCK | Cell Cycle |
| S1476 | SB525334 | 343.42 | 356559-20-1 | TGF-beta/Smad | TGF-beta/Smad |
| S1481 | Zosuquidar (LY335979) 3HCl | 636.99 | 167465-36-3 | P-gp | Transmembrane Transporters |
| S1487 | PHA-793887 | 361.48 | 718630-59-2 | CDK | Cell Cycle |
| S1489 | PIK-93 | 389.88 | 593960-11-3 | PI3K | PI3K/Akt/mTOR |
| S1490 | Ponatinib (AP24534) | 532.56 | 943319-70-8 | PDGFR,FGFR,VEGFR,Bcr-Abl | Angiogenesis |
| S1494 | LY2228820 | 612.74 | 862507-23-1 | p38 MAPK | MAPK |
| S1501 | Mycophenolate Mofetil | 433.49 | 128794-94-5 | Dehydrogenase | Metabolism |
| S1522 | Tosedostat (CHR2797) | 406.47 | 238750-77-1 | Aminopeptidase | Metabolism |
| S1528 | LY2811376 | 320.36 | 1194044-20-6 | Beta Amyloid,Gamma-secretase | Proteases |
| S1533 | R406 (free base) | 470.45 | 841290-80-0 | Syk | Angiogenesis |
| S1536 | CP-673451 | 417.50 | 343787-29-1 | PDGFR | Protein Tyrosine Kinase |
| S1537 | DMXAA (Vadimezan) | 282.29 | 117570-53-3 | VDA | Angiogenesis |
| S1548 | Dapagliflozin | 408.87 | 461432-26-8 | SGLT | GPCR & G Protein |
| S1555 | AZD8055 | 465.54 | 1009298-09-2 | mTOR | PI3K/Akt/mTOR |
| S1558 | AT7867 | 337.85 | 857531-00-1 | S6 Kinase,Akt | PI3K/Akt/mTOR |
| S1561 | BMS-777607 | 512.89 | 1025720-94-8 | Axl,c-Met | Protein Tyrosine Kinase |
| S1567 | Pomalidomide | 273.24 | 19171-19-8 | TNF-alpha | Apoptosis |
| S1570 | KU-60019 | 547.67 | 925701-49-1 | ATM/ATR | DNA Damage |
| S1577 | Tie2 kinase inhibitor | 439.53 | 948557-43-5 | Tie-2 | Protein Tyrosine Kinase |
| S1593 | Apixaban | 459.50 | 503612-47-3 | Factor Xa | Metabolism |
| S1630 | Allopurinol | 136.11 | 315-30-0 | OX Receptor | GPCR & G Protein |
| S1657 | Enalaprilat Dihydrate | 348.40 | 84680-54-6 | RAAS | Endocrinology & Hormones |
| S1703 | Divalproex Sodium | 310.41 | 76584-70-8 | Autophagy | Ubiquitin |
| S1802 | Acadesine | 258.23 | 2627-69-2 | AMPK | PI3K/Akt/mTOR |
| S1835 | Azithromycin | 748.98 | 83905-01-5 | Autophagy | Ubiquitin |
| S1909 | Fluvastatin Sodium | 433.45 | 93957-55-2 | HMG-CoA Reductase | Metabolism |
| S1983 | Adenine HCl | 171.59 | 2922-28-3 | DNA/RNA Synthesis | DNA Damage |
| S2013 | PF-573228 | 491.49 | 869288-64-2 | FAK | Angiogenesis |
| S2029 | Uridine | 244.20 | 58-96-8 | DNA/RNA Synthesis | DNA Damage |
| S2079 | Moexipril HCl | 535.03 | 82586-52-5 | RAAS | Endocrinology & Hormones |
| S2149 | GSK1292263 | 456.56 | 1032823-75-8 | GPR | Endocrinology & Hormones |
| S2151 | LDE225 (NVP-LDE225,Erismodegib) | 485.50 | 956697-53-3 | Hedgehog/Smoothened | Stem Cells & Wnt |
| S2153 | CGS 21680 HCl | 535.98 | 124431-80-7 | 5-alpha Reductase | Angiogenesis |
| S2169 | Rosuvastatin Calcium | 500.57 | 147098-20-2 | RAAS | Endocrinology & Hormones |
| S2170 | Givinostat (ITF2357) | 475.97 | 732302-99-7 | HDAC | Cytoskeletal Signaling |
| S2178 | AG-14361 | 320.39 | 328543-09-5 | PARP | DNA Damage |
| S2179 | LY2784544 | 469.94 | 1229236-86-5 | JAK | JAK/STAT |
| S2198 | SGI-1776 free base | 405.42 | 1025065-69-3 | Pim | JAK/STAT |
| S2199 | Aliskiren Hemifumarate | 609.83 | 173334-58-2 | RAAS | Endocrinology & Hormones |
| S2207 | PIK-293 | 397.43 | 900185-01-5 | PI3K | PI3K/Akt/mTOR |
| S2214 | AZ 960 | 354.36 | 905586-69-8 | JAK | JAK/STAT |
| S2219 | CYT387 | 414.46 | 1056634-68-4 | JAK | JAK/STAT |
| S2224 | UK 383367 | 324.38 | 348622-88-8 | Procollagen C Proteinase | Metabolism |
| S2225 | TAME | 342.41 | 901-47-3 | E3 Ligase ,APC | Cell Cycle |
| S2228 | VX-765 | 509.00 | 273404-37-8 | Caspase | Apoptosis |
| S2233 | Esomeprazole Sodium | 367.40 | 161796-78-7 | ATPase | Transmembrane Transporters |
| S2247 | BKM120 (NVP-BKM120, Buparlisib) | 410.39 | 944396-07-0 | PI3K | PI3K/Akt/mTOR |
| S2341 | (-)-Parthenolide | 248.32 | 20554-84-1 | E3 Ligase | Ubiquitin |
| S2449 | Forskolin | 410.50 | 66575-29-9 | cAMP | GPCR & G Protein |
| S2603 | Tioxolone | 168.17 | 4991-65-5 | Carbonic Anhydrase | Metabolism |
| S2621 | AZD5438 | 371.46 | 602306-29-6 | CDK | Cell Cycle |
| S2626 | LY2603618 | 436.30 | 911222-45-2 | Chk | Cell Cycle |
| S2631 | URB597 | 338.40 | 546141-08-6 | FAAH | Metabolism |
| S2632 | BMS-378806 | 406.43 | 357263-13-9 | gp120/CD4 | Others |
| S2662 | ICG-001 | 548.63 | 780757-88-2 | Wnt/beta-catenin | Stem Cells & Wnt |
| S2673 | Trametinib (GSK1120212) | 615.39 | 871700-17-3 | MEK | MAPK |
| S2674 | A922500 | 428.48 | 959122-11-3 | Transferase | Metabolism |
| S2680 | Ibrutinib (PCI-32765) | 440.50 | 936563-96-1 | BTK | Angiogenesis |
| S2694 | XL335 | 438.47 | 629664-81-9 | FXR | Others |
| S2704 | LY2109761 | 441.52 | 700874-71-1 | TGF-beta/Smad | TGF-beta/Smad |
| S2729 | SB415286 | 359.72 | 264218-23-7 | GSK-3 | PI3K/Akt/mTOR |
| S2738 | PAC-1 | 392.49 | 315183-21-2 | Caspase | Apoptosis |
| S2762 | Alectinib (CH5424802) | 482.62 | 1256580-46-7 | ALK | Protein Tyrosine Kinase |
| S2773 | SB705498 | 429.23 | 501951-42-4 | TRPV | Others |
| S2775 | Nocodazole | 301.32 | 31430-18-9 | Microtubule Associated,Autophagy | Cytoskeletal Signaling |
| S2777 | PF-5274857 | 436.96 | 1373615-35-0 | Hedgehog/Smoothened | Stem Cells & Wnt |
| S2780 | I-BET151 (GSK1210151A) | 415.44 | 1300031-49-5 | Epigenetic Reader Domain | Epigenetics |
| S2791 | Sotrastaurin | 438.48 | 425637-18-9 | PKC | TGF-beta/Smad |
| S2792 | Torcetrapib | 600.47 | 262352-17-0 | CETP | Metabolism |
| S2797 | Lonafarnib | 638.82 | 193275-84-2 | Transferase | Metabolism |
| S2803 | Galeterone | 388.55 | 851983-85-2 | P450 (e.g. CYP17),Androgen Receptor | Endocrinology & Hormones |
| S2804 | Sirtinol | 394.47 | 410536-97-9 | Sirtuin | Epigenetics |
| S2807 | Dabrafenib (GSK2118436) | 519.56 | 1195765-45-7 | Raf | MAPK |
| S2808 | GDC-0068 | 458.00 | 1001264-89-6 | Akt | PI3K/Akt/mTOR |
| S2811 | INK 128 (MLN0128) | 309.33 | 1224844-38-5 | mTOR | PI3K/Akt/mTOR |
| S2818 | CI994 (Tacedinaline) | 269.30 | 112522-64-2 | HDAC | Epigenetics |
| S2821 | RG108 | 334.33 | 48208-26-0 | Transferase,DNA Methyltransferase | Epigenetics |
| S2823 | Tideglusib | 334.39 | 865854-05-3 | GSK-3 | PI3K/Akt/mTOR |
| S2825 | ML133 HCl | 313.82 | 1222781-70-5 | Potassium Channel | Transmembrane Transporters |
| S2842 | SAR131675 | 358.39 | 1433953-83-3 | VEGFR | Protein Tyrosine Kinase |
| S2843 | BI-D1870 | 391.42 | 501437-28-1 | S6 Kinase | PI3K/Akt/mTOR |
| S2857 | MK-801 (Dizocilpine) | 221.30 | 77086-21-6 | GluR | Neuronal Signaling |
| S2859 | Golvatinib (E7050) | 633.69 | 928037-13-2 | VEGFR,c-Met | Protein Tyrosine Kinase |
| S2868 | Alogliptin | 339.39 | 850649-61-5 | DPP-4 | Proteases |
| S2903 | Lumiracoxib | 293.72 | 220991-20-8 | COX | Neuronal Signaling |
| S2907 | Pirfenidone | 185.22 | 53179-13-8 | TGF-beta/Smad | TGF-beta/Smad |
| S2912 | WZ811 | 290.36 | 55778-02-4 | CXCR | GPCR & G Protein |
| S2915 | GW9662 | 276.68 | 22978-25-2 | PPAR | DNA Damage |
| S2927 | Apoptosis Activator 2 | 306.14 | 79183-19-0 | Caspase | Apoptosis |
| S3008 | Zaltoprofen | 298.36 | 74711-43-6 | COX | Neuronal Signaling |
| S4014 | Hyoscyamine | 289.37 | 101-31-5 | AChR | Neuronal Signaling |
| S4021 | Tolcapone | 273.24 | 134308-13-7 | Transferase | Metabolism |
| S4039 | Methazolamide | 236.27 | 554-57-4 | Carbonic Anhydrase | Metabolism |
| S4075 | Zinc Pyrithione | 317.70 | 13463-41-7 | Proton Pump | Transmembrane Transporters |
| S4079 | Ticagrelor | 522.57 | 274693-27-5 | P2 Receptor | Neuronal Signaling |
| S4081 | Sulfacetamide Sodium | 236.22 | 127-56-0 | Autophagy | Ubiquitin |
| Catalog # | Compound Name | M.W. | CAS # | Targets | Pathway |
| --- | --- | --- | --- | --- | --- |
| S7004 | EPZ005687 | 539.67 | 1396772-26-1 | Histone Methyltransferase | Epigenetics |
| S7010 | GDC-0152 | 498.64 | 873652-48-3 | IAP | Apoptosis |
| S7018 | CZC24832 | 364.40 | 1159824-67-5 | PI3K | PI3K/Akt/mTOR |
| S7070 | GSK J4 HCl | 453.96 | 1373423-53-0 | Histone demethylases | Epigenetics |
| S7096 | KY02111 | 376.86 | 1118807-13-8 | Wnt/beta-catenin | Stem Cells & Wnt |
| S7129 | PYR-41 | 371.30 | 418805-02-4 | E1 Activating | Ubiquitin |
| S7133 | P22077 | 315.32 | 1247819-59-5 | DUB | Ubiquitin |
| S7134 | IU1 | 300.37 | 314245-33-5 | DUB | Proteases |
| S7140 | TCID | 283.92 | 30675-13-9 | DUB | Ubiquitin |
| S7155 | Batimastat (BB-94) | 477.64 | 130370-60-4 | MMP | Proteases |
| S7162 | Mdivi-1 | 353.22 | 338967-87-6 | Dynamin | Cytoskeletal Signaling |
| S7185 | AGI-5198 | 462.56 | 1355326-35-0 | Dehydrogenase | Metabolism |
| S7195 | RKI-1447 | 326.37 | 1342278-01-6 | ROCK | Cell Cycle |
| S7199 | DBeQ | 340.42 | 177355-84-9 | p97 | Ubiquitin |
| S7214 | Skepinone-L | 425.42 | 1221485-83-1 | p38 MAPK | MAPK |
| S7242 | Erastin | 547.04 | 571203-78-6 | Ferroptosis | Metabolism |
| S7243 | Ferrostatin-1 (Fer-1) | 262.35 | 347174-05-4 | Ferroptosis | Metabolism |
| S7265 | MM-102 | 669.80 | 1417329-24-8 | Histone Methyltransferase | Epigenetics |
| S7266 | Golgicide A | 284.30 | 1139889-93-2 | ATPase | Transmembrane Transporters |
| S7276 | SGI-1027 | 461.52 | 1020149-73-8 | DNA Methyltransferase | Epigenetics |
| S7279 | Suvorexant (MK-4305) | 450.92 | 1030377-33-3 | OX Receptor | GPCR & G Protein |
| S7319 | EHop-016 | 430.55 | 1380432-32-5 | Rac | Cell Cycle |
| S7326 | Tasisulam | 415.11 | 519055-62-0 | Caspase | Apoptosis |
| S8015 | CEP-32496 | 517.46 | 1188910-76-0 | CSF-1R,Raf | MAPK |
| S8016 | TAK-438 | 461.46 | 1260141-27-2 | Potassium Channel | Transmembrane Transporters |
| S8020 | GW0742 | 471.49 | 317318-84-6 | PPAR | Metabolism |
| S8021 | Vortioxetine (Lu AA21004) HBr | 379.36 | 960203-27-4 | 5-HT Receptor | Neuronal Signaling |
| S8022 | Empagliflozin (BI 10773) | 450.91 | 864070-44-0 | SGLT | GPCR & G Protein |
| S8028 | Tariquidar | 646.73 | 206873-63-4 | P-gp | Transmembrane Transporters |
| S8044 | BMS-345541 | 255.32 | 445430-58-0 | IκB/IKK | NF-κB |
| S8048 | ABT-199 (GDC-0199) | 868.44 | 1257044-40-8 | Bcl-2 | Apoptosis |
| S8051 | Macitentan | 588.27 | 441798-33-0 | Endothelin Receptor | GPCR & G Protein |
| S8058 | P276-00 | 438.30 | 920113-03-7 | CDK | Cell Cycle |
| S1021 | Dasatinib | 488.01 | 302962-49-8 | Bcr-Abl,c-Kit,Src | Angiogenesis |
| S1143 | AG-490 (Tyrphostin B42) | 294.3 | 133550-30-8 | JAK,EGFR | Protein Tyrosine Kinase |
| S2867 | WHI-P154 | 376.2 | 211555-04-3 | JAK,EGFR | JAK/STAT |
| S7152 | C646 | 445.42 | 328968-36-1 | Histone Acetyltransferase | Epigenetics |
| S7256 | SGC-CBP30 | 509.04 | N/A | Epigenetic Reader Domain | Epigenetics |
| S7360 | OTX015 | 491.99 | 202590-98-5 | BET | Epigenetics |
| S7373 | UNC669 | 338.24 | 1314241-44-5 | MBT | Epigenetics |
| S1042 | Sunitinib Malate | 532.56 | 341031-54-7 | VEGFR,PDGFR,c-Kit | Protein Tyrosine Kinase |
| S1088 | NVP-ADW742 | 453.58 | 475488-23-4 | IGF-1R | Protein Tyrosine Kinase |
| S2134 | AZD8330 | 461.23 | 869357-68-6 | MEK | MAPK |
| S2162 | AZD1480 | 348.77 | 935666-88-9 | JAK | |
| S2619 | MG-132 | 475.62 | 133407-82-6 | Proteasome | Proteases |
| S2633 | NPS-2143 | 408.92 | 284035-33-2 | CaSR | GPCR & G Protein |
| S2658 | GSK2126458 (GSK458) | 505.50 | 1086062-66-9 | PI3K,mTOR | PI3K/Akt/mTOR |
| S2672 | PF-00562271 | 665.66 | 939791-38-5 | FAK | Angiogenesis |
| S2759 | CUDC-907 | 508.55 | 1339928-25-4 | PI3K,HDAC | Cytoskeletal Signaling |
| S2769 | Dovitinib (TKI-258) Dilactic Acid | 572.59 | 852433-84-2 | PDGFR,FGFR,c-Kit,FLT3,VEGFR | Angiogenesis |
| S2801 | AZD4547 | 463.57 | 1035270-39-3 | FGFR | Angiogenesis |
| S2809 | MPEP | 193.24 | 96206-92-7 | GluR | Neuronal Signaling |
| S2817 | Torin 2 | 432.40 | 1223001-51-1 | ATM/ATR,mTOR | PI3K/Akt/mTOR |
| S2827 | Torin 1 | 607.62 | 1222998-36-8 | Autophagy,mTOR | PI3K/Akt/mTOR |
| S2847 | Cathepsin Inhibitor 1 | 401.89 | 225120-65-0 | Others | Others |
| S2849 | SB269970 HCl | 388.95 | 261901-57-9 | 5-HT Receptor | Neuronal Signaling |
| S2890 | PF-562271 | 507.49 | 717907-75-0 | FAK | Angiogenesis |
| S2891 | GW441756 | 275.30 | 504433-23-2 | Trk receptor | Protein Tyrosine Kinase |
| S2898 | MLN0905 | 486.56 | 1228960-69-7 | PLK | |
| S2906 | Ki16198 | 488.98 | 355025-13-7 | LPA Receptor | GPCR & G Protein |
| S2919 | IOX2 | 352.34 | 931398-72-0 | HIF | Angiogenesis |
| S3051 | Bosentan Hydrate | 569.63 | 157212-55-0 | Endothelin Receptor | GPCR & G Protein |
| S3603 | Betulinic acid | 456.70 | 472-15-1 | Topoisomerase | DNA Damage |
| S4001 | Cabozantinib malate (XL184) | 635.59 | 1140909-48-3 | Axl,VEGFR | |
| S4017 | Allylthiourea | 116.18 | 109-57-9 | Others | Others |
| S4019 | Avanafil | 483.95 | 330784-47-9 | PDE | Metabolism |
| S4035 | Vitamin D2 | 396.65 | 50-14-6 | Others | Others |
| S4063 | Vitamin D3 | 384.64 | 67-97-0 | Others | Others |
| S4083 | Vitamin A Acetate | 328.49 | 127-47-9 | Others | |
| S4245 | Sodium ascorbate | 201.13 | 134-03-2 | Others | Others |
| S4901 | JNK-IN-8 | 507.59 | 1410880-22-6 | JNK | |
| S6002 | NXY-059 | 381.33 | 168021-79-2 | Others | Others |
| S7047 | BAY 1000394 | 430.44 | 1223498-69-8 | CDK | |
| S7060 | PP1 | 281.36 | 172889-26-8 | Src | Angiogenesis |
| S7064 | FMK | 342.37 | 821794-92-7 | S6 Kinase | |
| S7077 | Cilengitide | 588.66 | 188968-51-6 | Integrin | Others |
| S7087 | GSK2334470 | 462.59 | 1227911-45-6 | PDK-1 | |
| S7099 | (-)-Blebbistatin | 292.33 | 856925-71-8 | ATPase | Transmembrane Transporters |
| S7101 | SCH772984 | 587.67 | 942183-80-4 | ERK | |
| S7103 | GDC-0032 | 460.53 | 1282512-48-4 | PI3K | |
| S7118 | AZD3839 | 449.40 | 1227163-56-5 | BACE | |
| S7120 | 3-Deazaneplanocin A (DZNeP) | 262.26 | 102052-95-9 | Histone Methyltransferase | Epigenetics |
| S7167 | SSR128129E | 346.31 | 848318-25-2 | FGFR | Angiogenesis |
| S7253 | AZD2858 | 453.52 | 486424-20-8 | GSK-3 | PI3K/Akt/mTOR |
| S7280 | Edoxaban | 548.06 | 480449-70-5 | Factor Xa | Metabolism |
| S7338 | AZ191 | 429.52 | N/A | Others | Others |
| S7351 | JSH-23 | 240.34 | 749886-87-1 | NF-κB | NF-κB |
| S8000 | Tenovin-1 | 369.48 | 380315-80-0 | p53,E3 Ligase | Apoptosis |
| S8032 | PRT062607 (P505-15, BIIB057) HCl | 429.91 | 1370261-97-4 | Syk | Angiogenesis |
| S8034 | Apremilast (CC-10004) | 460.50 | 608141-41-9 | PDE | Metabolism |
| S8035 | VU 0364439 | 422.29 | 1246086-78-1 | GluR | Neuronal Signaling |
| S8056 | Lomeguatrib | 326.17 | 192441-08-0 | DNA Methyltransferase | Epigenetics |
| S8057 | Pacritinib (SB1518) | 472.58 | 937272-79-2 | JAK | JAK/STAT |
| S8061 | Sabutoclax | 700.78 | 1228108-65-3 | Bcl-2 | |
| S8078 | Bardoxolone Methyl | 505.69 | 218600-53-4 | IκB/IKK | NF-κB |
| S7438 | ME0328 | 321.37 | 1445251-22-8 | PARP | DNA Damage |
| S8005 | SMI-4a | 273.23 | 438190-29-5 | Pim | JAK/STAT |
| S1012 | BMS-536924 | 479.96 | 468740-43-4 | IGF-1R | |
| S1091 | OSI-906 (Linsitinib) | 421.49 | 867160-71-2 | IGF-1R | Protein Tyrosine Kinase |
| S1107 | Danusertib (PHA-739358) | 474.55 | 827318-97-8 | c-RET,FGFR,Bcr-Abl,Aurora Kinase | Cell Cycle |
| S1116 | Palbociclib (PD-0332991) HCl | 483.99 | 827022-32-2 | CDK | Cell Cycle |
| S1121 | TW-37 | 573.70 | 877877-35-5 | Bcl-2 | Apoptosis |
| S1133 | Alisertib (MLN8237) | 518.92 | 1028486-01-2 | Aurora Kinase | Cell Cycle |
| S1134 | AT9283 | 381.43 | 896466-04-9 | JAK,Aurora Kinase,Bcr-Abl | JAK/STAT |
| S1142 | 17-DMAG (Alvespimycin) HCl | 653.21 | 467214-21-7 | HSP (e.g. HSP90) | Cytoskeletal Signaling |
| S1159 | Ganetespib (STA-9090) | 364.40 | 888216-25-9 | HSP (e.g. HSP90) | Cytoskeletal Signaling |
| S1172 | JNJ-26854165 (Serdemetan) | 328.41 | 881202-45-5 | p53,E3 Ligase | Apoptosis |
| S1176 | Plinabulin (NPI-2358) | 336.39 | 714272-27-2 | VDA | |
| S1334 | Flupirtine maleate | 420.39 | 75507-68-5 | DNA/RNA Synthesis | DNA Damage |
| S1366 | BMS-707035 | 410.42 | 729607-74-3 | Integrase | Microbiology |
| S1369 | Bafetinib (INNO-406) | 576.62 | 859212-16-1 | Bcr-Abl | |
| S1384 | Mizoribine | 259.22 | 50924-49-7 | DNA/RNA Synthesis | DNA Damage |
| S1392 | Pelitinib (EKB-569) | 467.92 | 257933-82-7 | EGFR | Protein Tyrosine Kinase |
| S1413 | Bafilomycin A1(Baf-A1) | 622.83 | 88899-55-2 | Proton Pump | |
| S1451 | Aurora A Inhibitor I | 588.07 | 1158838-45-9 | Aurora Kinase | Cell Cycle |
| S1454 | PHA-680632 | 501.62 | 398493-79-3 | Aurora Kinase | Cell Cycle |
| S1458 | VX-745 | 436.26 | 209410-46-8 | p38 MAPK | MAPK |
| S1470 | TSU-68 (SU6668, Orantinib) | 310.35 | 252916-29-3 | VEGFR,PDGFR,FGFR | Protein Tyrosine Kinase |
| S1473 | GW501516 | 453.50 | 317318-70-0 | PPAR | |
| S1478 | Oligomycin A | 791.06 | 579-13-5 | ATPase | Transmembrane Transporters |
| S1519 | CCT129202 | 497.02 | 942947-93-5 | Aurora Kinase | Cell Cycle |
| S1525 | MK-1775 | 500.60 | 955365-80-7 | Wee1 | Cell Cycle |
| S1526 | Quizartinib (AC220) | 560.67 | 950769-58-1 | FLT3 | Angiogenesis |
| S1541 | EX 527 (Selisistat) | 248.71 | 49843-98-3 | Sirtuin | Epigenetics |
| S1550 | Pimobendan | 334.37 | 74150-27-9 | PDE | Metabolism |
| S1556 | PHT-427 | 409.61 | 1191951-57-1 | PDK-1,Akt | PI3K/Akt/mTOR |
| S1572 | BS-181 HCl | 416.99 | 1397219-81-6 | CDK | Cell Cycle |
| S1574 | BIRB 796 (Doramapimod) | 527.66 | 285983-48-4 | p38 MAPK | MAPK |
| S1679 | Flurbiprofen | 244.26 | 5104-49-4 | Others | Others |
| S2001 | Elvitegravir (GS-9137, JTK-303) | 447.88 | 697761-98-1 | Integrase | Microbiology |
| S2017 | Benidipine HCl | 542.02 | 91599-74-5 | Calcium Channel | Transmembrane Transporters |
| S2047 | Lornoxicam | 371.82 | 70374-39-9 | COX | Neuronal Signaling |
| S2077 | Atorvastatin Calcium | 1155.34 | 134523-03-8 | HMG-CoA Reductase | Metabolism |
| S2102 | Rasagiline Mesylate | 267.34 | 161735-79-1 | MAO | Metabolism |
| S2121 | Licofelone | 379.88 | 156897-06-2 | COX | Neuronal Signaling |
| S2150 | Neratinib (HKI-272) | 557.04 | 698387-09-6 | HER2,EGFR | Protein Tyrosine Kinase |
| S2158 | KW-2449 | 332.40 | 1000669-72-6 | Aurora Kinase,Bcr-Abl,FLT3 | Angiogenesis |
| S2180 | MLN2238 | 361.03 | 1072833-77-2 | Proteasome | Proteases |
| S2181 | MLN9708 | 517.12 | 1201902-80-8 | Proteasome | Proteases |
| S2183 | BGJ398 (NVP-BGJ398) | 560.48 | 872511-34-7 | FGFR | Angiogenesis |
| S2185 | AST-1306 | 621.08 | 1050500-29-2 | EGFR | Protein Tyrosine Kinase |
| S2197 | A-966492 | 324.35 | 934162-61-5 | PARP | |
| S2201 | BMS-794833 | 468.84 | 1174046-72-0 | VEGFR,c-Met | Protein Tyrosine Kinase |
| S2216 | Mubritinib (TAK 165) | 468.47 | 366017-09-6 | HER2 | Protein Tyrosine Kinase |
| S2222 | PF-3716556 | 394.47 | 928774-43-0 | ATPase,Proton Pump | |
| S2227 | PIK-294 | 489.53 | 900185-02-6 | PI3K | PI3K/Akt/mTOR |
| S2235 | Volasertib (BI 6727) | 618.81 | 755038-65-4 | PLK | Cell Cycle |
| S2238 | Palomid 529 (P529) | 406.43 | 914913-88-5 | mTOR | PI3K/Akt/mTOR |
| S2623 | Omecamtiv mecarbil (CK-1827452) | 401.43 | 873697-71-3 | ATPase | Transmembrane Transporters |
| S2624 | OSI-027 | 406.44 | 936890-98-1 | mTOR | PI3K/Akt/mTOR |
| S2625 | Fostamatinib (R788) | 580.46 | 901119-35-5 | Syk | Angiogenesis |
| S2627 | Tubastatin A HCl | 371.86 | 1310693-92-5 | HDAC | Epigenetics |
| S2637 | TAK-875 | 533.63 | 1374598-80-7 | GPR | Endocrinology & Hormones |
| S2667 | Dolutegravir (GSK1349572) | 419.38 | 1051375-16-6 | Integrase | Microbiology |
| S2689 | WAY-600 | 494.59 | 1062159-35-6 | mTOR | PI3K/Akt/mTOR |
| S1028 | Lapatinib (GW-572016) Ditosylate | 925.46 | 388082-77-7 | HER2,EGFR | Protein Tyrosine Kinase |
| S1030 | Panobinostat (LBH589) | 349.43 | 404950-80-7 | HDAC | |
| S1205 | PIK-75 | 488.74 | 372196-77-5 | PI3K,DNA-PK | PI3K/Akt/mTOR |
| S1248 | Vinblastine | 810.97 | 865-21-4 | Microtubule Associated | Cytoskeletal Signaling |
| S1360 | GSK1059615 | 333.36 | 958852-01-2 | PI3K,mTOR | PI3K/Akt/mTOR |
| S2055 | Gimeracil | 145.54 | 103766-25-2 | Dehydrogenase | Metabolism |
| S2730 | Crenolanib (CP-868596) | 443.54 | 670220-88-9 | PDGFR | Protein Tyrosine Kinase |
| S2735 | MK-8776 (SCH 900776) | 376.25 | 891494-63-6 | CDK,Chk | Cell Cycle |
| S7138 | BMS-833923 | 473.57 | 1059734-66-5 | Hedgehog/Smoothened | GPCR & G Protein |
| S7311 | Q-VD-OPh | 513.49 | 1135695-98-5 | Caspase | |

## Slide 2
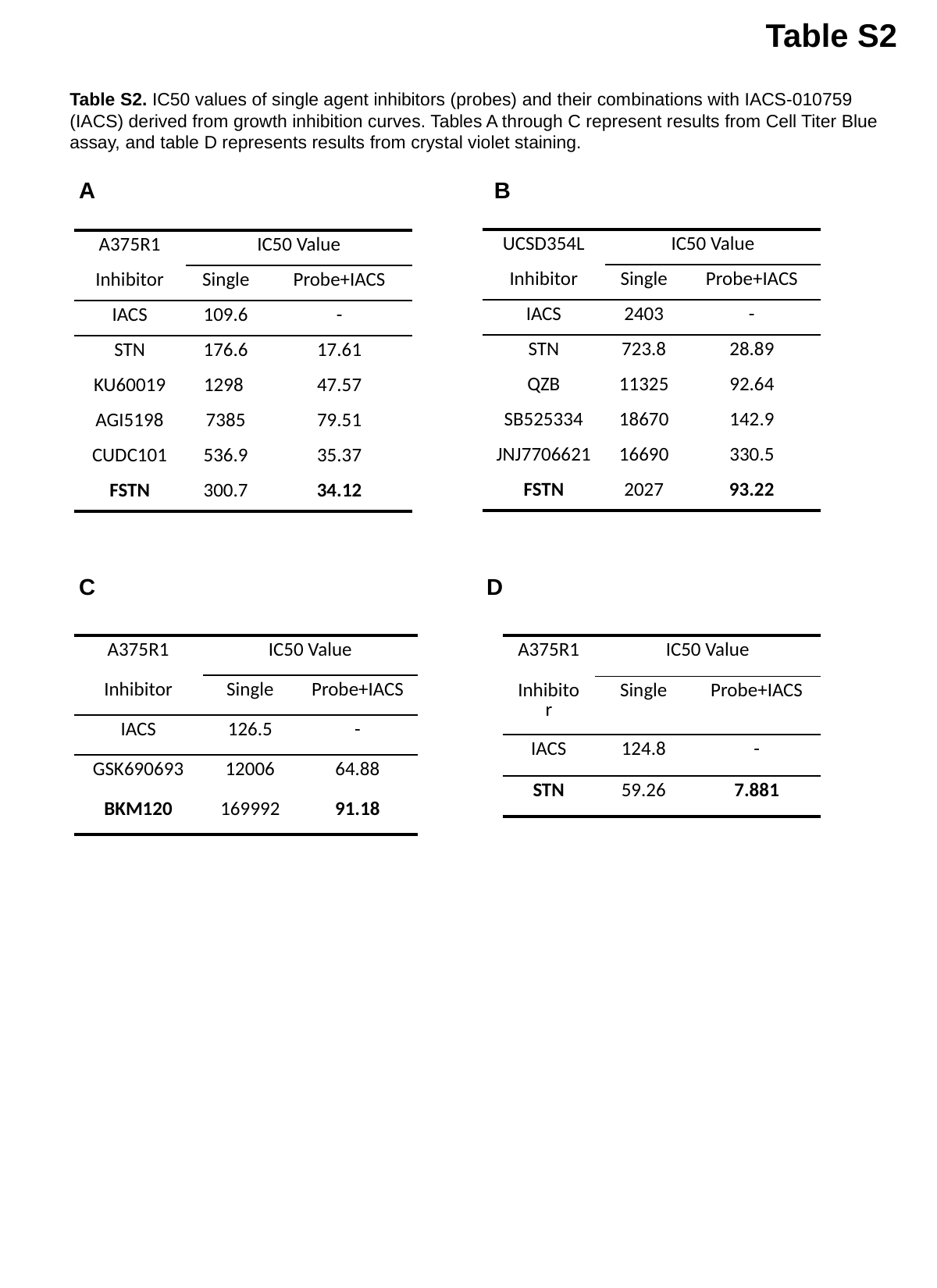

Table S2
Table S2. IC50 values of single agent inhibitors (probes) and their combinations with IACS-010759 (IACS) derived from growth inhibition curves. Tables A through C represent results from Cell Titer Blue assay, and table D represents results from crystal violet staining.
B
A
| UCSD354L | IC50 Value | |
| --- | --- | --- |
| Inhibitor | Single | Probe+IACS |
| IACS | 2403 | - |
| STN | 723.8 | 28.89 |
| QZB | 11325 | 92.64 |
| SB525334 | 18670 | 142.9 |
| JNJ7706621 | 16690 | 330.5 |
| FSTN | 2027 | 93.22 |
| A375R1 | IC50 Value | |
| --- | --- | --- |
| Inhibitor | Single | Probe+IACS |
| IACS | 109.6 | - |
| STN | 176.6 | 17.61 |
| KU60019 | 1298 | 47.57 |
| AGI5198 | 7385 | 79.51 |
| CUDC101 | 536.9 | 35.37 |
| FSTN | 300.7 | 34.12 |
C
D
| A375R1 | IC50 Value | |
| --- | --- | --- |
| Inhibitor | Single | Probe+IACS |
| IACS | 126.5 | - |
| GSK690693 | 12006 | 64.88 |
| BKM120 | 169992 | 91.18 |
| A375R1 | IC50 Value | |
| --- | --- | --- |
| Inhibitor | Single | Probe+IACS |
| IACS | 124.8 | - |
| STN | 59.26 | 7.881 |
